# Jasmonic Acid Plays a Pivotal Role in Pollen Development and Fertility Regulation in Different Types of P(T)GMS Rice Lines

**DOI:** 10.3390/ijms22157926

**Published:** 2021-07-25

**Authors:** Ying He, Chen Liu, Lan Zhu, Ming Fu, Yujun Sun, Hanlai Zeng

**Affiliations:** MOA Key Laboratory of Crop Ecophysiology and Farming System in the Middle Reaches of the Yangtze River, College of Plant Science and Technology, Huazhong Agricultural University, Wuhan 430070, China; yinghe@mail.hzau.edu.cn (Y.H.); lc812049@hotmail.com (C.L.); lanzhu@mail.hzau.edu.cn (L.Z.); fuming2016@webmail.hzau.edu.cn (M.F.); yujunsun@webmail.hzau.edu.cn (Y.S.)

**Keywords:** PTGMS, PGMS, fertility regulation, JA biosynthetic pathway, spraying MEJA, spraying SHAM

## Abstract

Two-line hybrid rice systems represent a new technical approach to utilizing the advantages of rice hybrids. However, the mechanism underlying the male sterile-line fertility transition in rice remains unclear. Peiai 64S (PA64S) is a photoperiod- and thermo-sensitive genic male sterile (PTGMS) line in which male sterility manifests at an average temperature above 23.5 °C under long-day (LD) conditions. Nongken 58S (NK58S) is a LD-sensitive genic male sterile (PGMS) rice that is sterile under LD conditions (above 13.75 h-day). In contrast, D52S is a short-day (SD)-PGMS line that manifests male sterility under SD conditions (below 13.5 h-day). In this study, we obtained fertile and sterile plants from all three lines and performed transcriptome analyses on the anthers of the plants. Gene ontology (GO) analysis suggested that the differentially expressed genes identified were significantly enriched in common terms involved in the response to jasmonic acid (JA) and in JA biosynthesis. On the basis of the biochemical and molecular validation of dynamic, tissue-specific changes in JA, indole-3-acetic acid (IAA) levels, gibberellin (GA) levels, and JA biosynthetic enzyme activities and expression, we proposed that JA could play a pivotal role in viable pollen production through its initial upregulation, constant fluctuation and leaf-spikelet signaling under certain fertility-inducing conditions. Furthermore, we also sprayed methyl jasmonate (MEJA) and salicylhydroxamic acid (SHAM) on the plants, thereby achieving fertility reversal in the PGMS lines NK58S and D52S, with 12.91–63.53% pollen fertility changes. Through qPCR and enzyme activity analyses, we identified two key enzymes—allene oxide synthase (AOS) and allene oxide cyclase (AOC)—that were produced and upregulated by 20–500-fold in PGMS in response to spraying; the activities of these enzymes reversed pollen fertility by influencing the JA biosynthetic pathway. These results provide a new understanding of hormone interactions and networks in male-sterile rice based on the role of JA that will help us to better understand the potential regulatory mechanisms of fertility development in rice in the future.

## 1. Introduction

Rice serves as a food source for nearly 50% of the global population. With the reduction in arable land and the increase in the population worldwide, hybrid rice breeding has greatly increased food production, with a 55% yield increase during recent decades [1]. Among hybrid rice breeding approaches, the two-line hybrid rice breeding system was initiated and developed through the utilization of photoperiod- and thermo-sensitive genic male sterile (PTGMS) lines; this approach has many advantages, including increased yields and optimized hybrid seed production [2,3]. The specific characteristics of PTGMS lines are reflected in the abnormal development of stamens in male reproductive organs after the correct recognition of stigma, leading to failed self-fertilization. The fertility regulation process is controlled by the photoperiod and temperature. Currently, many PTGMS rice varieties have been discovered or created, such as Nongken 58S (NK58S), Peiai 64S (PA64S), Annong S-1, Guangzhan 63S and Y58S [4,5,6,7]. Hundreds of PTGMS varieties come from parents’ gene sources of NK58S and Annong S-1. A number of these varieties are widely used, with a very large production area in China, such as PA64S, Guangzhan 63S and Y58S. Thus, it is necessary to study their male sterile-line fertility transition mechanism.

The male fertility mechanism of PTGMS rice has consistently remained a research focus. With regard to genetic analysis, some studies have been conducted on different PTGMS rice varieties. For example, in the first natural mutant of photoperiod-sensitive genic male sterile (PGMS) rice, NK58S, two loci of PMS1 and PMS2 related to fertility regulation were identified on chromosomes 3 and 7, respectively [8]. Subsequently, a number of candidate genes related to fertility were reported in PTGMS rice, such as *tms*1, *tms*2, *ptgms*2-1, and *p/tms*12-1, located on chromosome 11 [9,10,11,12,13]. A great deal of information related to genic male sterility has been revealed through reverse genetics, such as the identification of cytochrome P450 members (*OsCYP703A3* and *OsCYP704B2*), fatty acyl-CoA synthetase (*OsACOS12*), polyketide synthases (*OsPKS1/2*), ATP binding cassette G transporters (*OsABCG15/26*), lipid transporter genes (*OsC6*) and 12-oxophytodeinoate reductase (*OsOPR7*) involved in lipid synthesis [14,15,16,17,18,19,20,21,22,23]. The pollen exine has a multi-layered structure, and the main component is sporopollenin. Sporopollenin has stable chemical properties and is synthesized from aliphatic derivatives and phenolic compounds in the tapetum. During the later stages of anther development, the tapetum layer degenerates and releases the lipids or phenols needed for normal anther development. Despite the conservative nature of the process of fatty acid synthesis, our research also reported that the timing of the tapetum degradation and substance release affect PA64S fertility [24].

Given the current developments in genome-wide sequencing technology, a very large number of microRNAs (miRNAs), lncRNAs and circRNAs involved in fertility transition and abiotic stress responses have been identified in plants. For example, miRNA expression profiles from rice anthers at high and low temperatures were also reported in PTGMS rice, which provides a beneficial pathway for understanding the role of miRNAs in rice anther and PTGMS development [25,26]. One key issue with the two-line system has been revealed as research on this topic has continued: the critical male-sterile temperature changes and shifts slightly with the increase in the number of generations produced, leading to substantial reproductive difficulties and causing uncertainties related to food security [27]. Studying the mechanisms of rice sterility and its regulation is, therefore, helpful for understanding the sterility system.

Jasmonic acid (JA) and its derivatives can affect aspects of plant development, signaling and defense, as well as viable pollen production, in particular [28,29,30]. The JA synthesis pathways in plants have been revealed, and four main enzymes in these pathways have been successfully identified. JA and its methyl ester, methyl jasmonate (MEJA), are derived from linolenic acid and form cyclopentanone-based compounds [31]. At the upstream stage of JA biosynthesis, DEFECTIVE IN ANTHER DEHISCENCE 1 (DAD1), located in chloroplasts, hydrolyses the triunsaturated fatty acids in glycerides and phospholipids to produce free α-linolenic acid [32]. Subsequently, three enzymes participate in three sequential steps: 13-lipoxygenases (13-LOXs), allene oxide synthase (AOS) and allene oxide cyclase (AOC) [33,34,35,36]. The product of these steps, (9S,13S)-12-oxo-phytodienoic acid (OPDA) is generated at the intermediate stage of the pathway. In peroxisomes, JA is synthesized by 12-oxophytodeinoate reductase (OPR) and β-oxidation cycles at the downstream stage [37]. Then, JA derivatives are formed through methylation and connected by amino acids. JA is synthesized in these plants in cells containing chloroplasts, where it can be sequestered as it is produced. However, the level of JA in plants varies as a function of the tissue and cell type and developmental stage, as well as in response to several different environmental stimuli [28,29,30,36]. These important functions of JA are closely related to its homeostasis and biosynthesis in plants. Some JA biosynthetic genes have been reported to play important roles in pollen development. For example, the male-sterile Arabidopsis and rice mutants *dad1* and *opr3/7* showed defects in anther dehiscence, pollen maturation, filament elongation, flower opening and viable pollen production [23,37,38]. The defects were rescued by the exogenous application of JA or linolenic acid [23,37].

Exogenous JA causes large and transient changes in the concentration of JA in most plant cells before reaching an internal equilibrium in tissues. However, few studies focus on the key role of JA in male sterility regulation in PTGMS rice, or the effect of exogenous JA spraying on regulating the fertility of PA64S and NK58S. In this study, we used fertile and sterile plants from one PTGMS and two PGMS rice types, PA64S, NK58S and D52S, respectively, to study the JA levels and JA biosynthetic processes in the anthers of fertile and sterile plants through transcriptome, biochemical and molecular analyses. We also performed MEJA and salicylhydroxamic acid (SHAM) spraying treatments to investigate whether JA plays a pivotal role in viable pollen production. We found that the JA level and the JA biosynthetic pathway plays a pivotal role in rice pollen fertility. It provides a new understanding of JA signaling and regulation in male-sterile rice in order to better understand its complex regulatory mechanisms.

## 2. Results

### 2.1. Photoperiod and Temperature Experimental System for Pollen Fertility Transformations of Both PGMS and PTGMS Rice Lines

For both PGMS and PTGMS rice lines, the critical stage for their male fertility transformation is from the 3rd to 7th stages, according to the eight-stage differentiation of young spikes [39]. The short-day/long-day (SD/LD)-PGMS D52S and NK58S rice required different critical day lengths for normal pollen development. To obtain sterile/fertile lines, we treated the plants with LD (14 h-light/10 h-dark) and SD (10 h-light/14 h-dark) cycles from 2017 to 2020. In the anthers of young NK58S panicles, few fertile pollen grains (4.63%) were detected under LD conditions, whereas 70.61% of the pollen grains were fertile under SD conditions (Table 1, Figure S1). In contrast, in D52S, 48.93% of the pollen grains were fertile under LD conditions, and only 1.34% were fertile under SD conditions (Table 1, Figure S1). This result indicated that D52S was a reverse PGMS line, exhibiting the opposite fertility outcome of NK58S under the same photoperiod. Under LD conditions, the pollen fertility of NK58S could be considered to indicate relative male sterility (called NK58S-S), and that of D52S could be considered to indicate partially induced fertility (called D52S-F). Under SD conditions, the pollen of NK58S was partially fertile (NK58S-F) and that of D52S was male sterile (D52S-S).

PA64S is a PTGMS rice line with a critical temperature for male sterility of 23.5 °C [6]. We treated PA64S plants with a high temperature (HT) of 28 °C and a low temperature (LT) of 21 °C to obtain sterile and fertile lines, respectively. Under HT, no fertile pollen grains (0.00%) were detected in the anthers of the young panicles, whereas 41.35% pollen fertility was detected under LT conditions (Table 1, Figure S1). This result indicated that the pollen of PA64S was partially fertile, called PA64S-F, when induced at a relative LT below the critical temperature of 23.5 °C. Above this temperature, the PA64S line showed complete male sterility (PA64S-S).

Among these three cultivars, the sterile and fertile lines displayed similar vegetative and non-reproductive floral organ morphologies, except that the sterile lines had slightly smaller anthers with mature pollen grains. The results suggested that our photoperiodic and temperature treatments were effective for fertility regulation and validated our experimental system.

### 2.2. JA regulates the Pollen Fertility of Both PGMS and PTGMS Rice Lines

**Figure 1:**
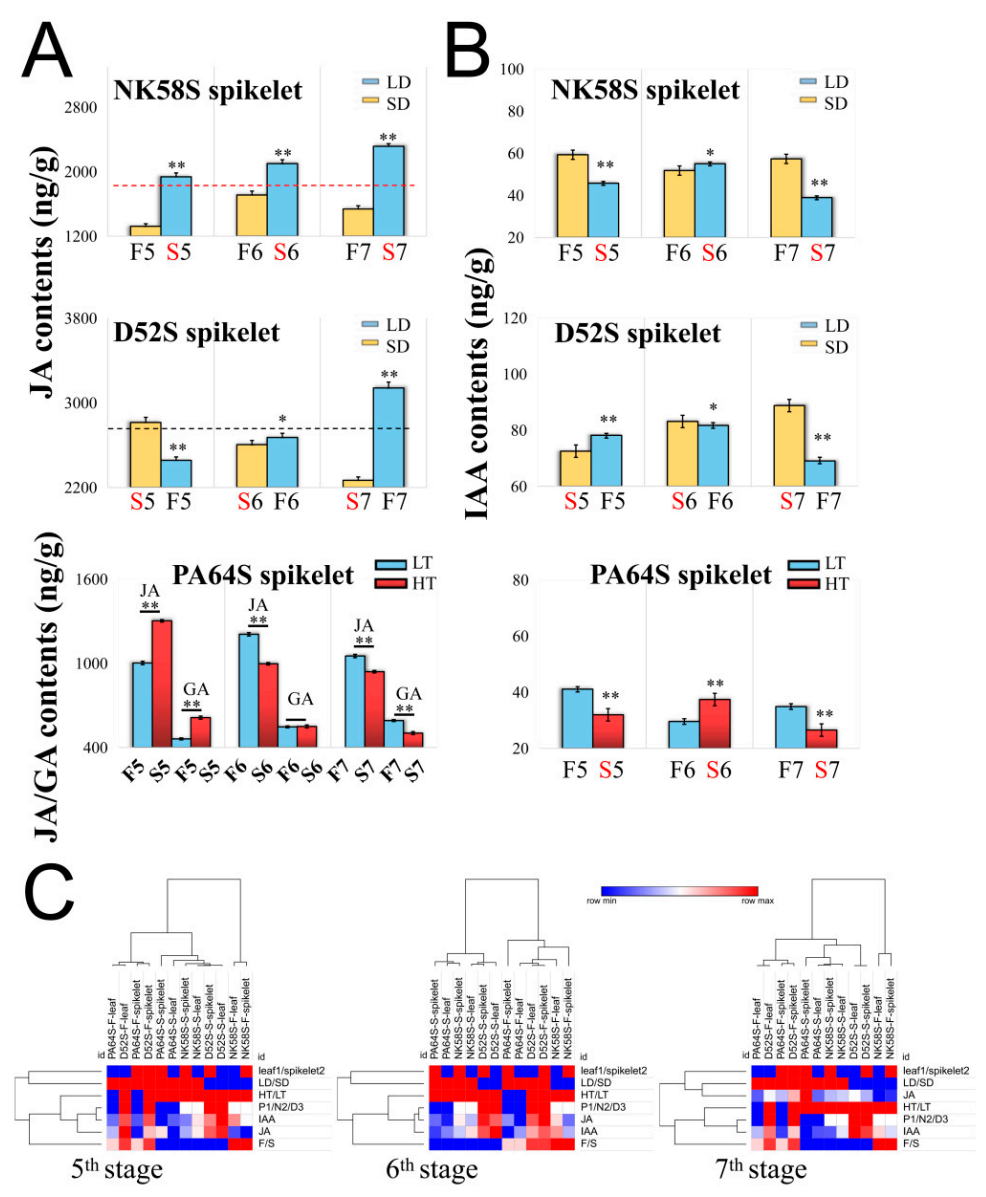
Analysis of hormone contents in D52S, NK58S and PA64S by photoperiod and temperature treatment. (**A**) JA and GA contents of spikelets from D52S, NK58S and PA64S treated by LD/SD and HT/LT. (**B**) IAA contents of spikelets from D52S, NK58S and PA64S. (**C**) Heatmap analysis of their hormone contents from 5th to 7th stages. The contents from HT/LT and LD/SD treated plants are columnar with red/blue and blue/yellow color, respectively. Data are shown as mean ± standard deviation (*n* = 3). Asterisks indicate significant differences between sterile and fertile plants revealed by Student’s *t*-test (*, *p* < 0.05; **, *p* < 0.01) for each line at the same stages. LD, long-day cycle; SD, short-day cycle; HT, high temperature; LT, low temperature; S/F, sterile/fertile plants; 5/6/7, 5th/6th/7th-stage; JA, jasmonic acid; IAA, indole-3-acetic acid; GA, gibberellin.

To investigate whether JA regulates rice pollen development, we measured the JA levels in young spikelets and sword leaves during the different reproductive stages of the sterile and fertile plants induced by the photoperiod or temperature. The baseline JA levels in the tissues varied among the different P(T)GMS lines. The JA contents in the tissues of D52S in general were slightly higher than those in the tissues of NK58S at all stages (Figure 1A and Figure S2). For PA64S, the change in the spikelet JA content was most significant at the 7th stage (Figure 1A). In terms of the differences in the JA content between sterile and fertile plants, the JA content in the spikelets of sterile plants was higher than that in the spikelets of fertile plants in the early stage of anther development (the 5th stage) in all three rice lines. In the 6th stage, the difference in JA content between the PGMS and PTGMS rice lines was not obvious. During subsequent development (the 7th stage), the JA contents of both the young spikelets and sword leaves of sterile plants were also higher than those of the fertile plants, except in the D52S spikelets (Figure 1A and Figure S2). During the same period, we also determined the indole-3-acetic acid (IAA) and gibberellin (GA) contents of young spikelets and sword leaves. The results were consistent for the P(T)GMS lines in that the IAA contents of the spikelets of the fertile plants were significantly higher than those of the spikelets of the sterile plants in the 5th stage (Figure 1B).

For PTGMS rice, the fertility of the rice pollen in PA64S was upregulated under LT condition. In contrast to the IAA trend, the GA level in the spikelets of PA64S-F was significantly lower than that in the spikelets of PA64S-S in the 5th stage (Figure 1 and Figure S2). These results indicated that other hormones in addition to JA mediate normal pollen development in the early stages of the pollen development process.

In PGMS rice, JA may be exchanged among tissues and participate in the regulation of the early stage of anther development. D52S and NK58S were opposing PGMS lines induced by the same photoperiod. Comparing the tissue JA content under LD and SD conditions revealed that the JA contents in spikelets under LD conditions were higher than those under SD conditions, except in the D52S line at the 5th stage (Figure 1A,C). For D52S and NK58S, the sword leaf JA contents under LD conditions were also higher than those under SD conditions in certain periods, for example, the 5th stage (Figure 1C and Figure S2). The tissue in the sword leaves is the most affected by the different photoperiods in the sensitive fertility period, and young spikelets are greatly affected by different photoperiods but also by different temperatures. These results indicated that the JA contents in rice tissues are upregulated by LD conditions, particularly the sword leaf JA content in the early stage of rice anther development.

### 2.3. Analysis of JA Synthetic Enzyme Activities in Pollen Fertility for Both PGMS and PTGMS Rice Lines

**Figure 2:**
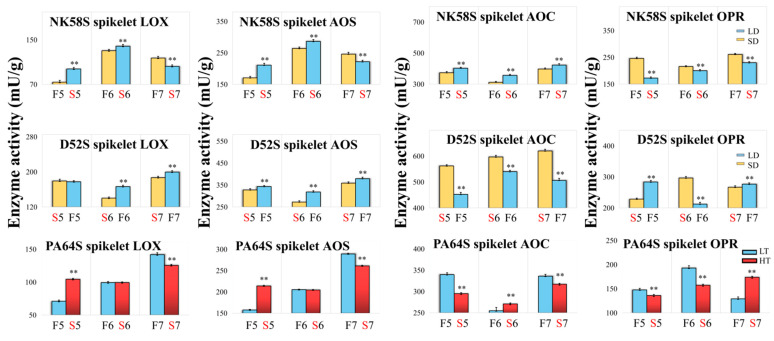
Four key enzyme activities of JA synthesis pathway in D52S, NK58S and PA64S spikelets by photoperiod and temperature treatment. The LD/SD and HT/LT treated plants are columnar with blue/yellow and red/blue color, respectively. Data are shown as mean ± standard deviation (*n* = 3). Asterisks indicate significant differences between sterile and fertile plants revealed by Student’s *t*-test (**, *p* < 0.01). LD, long-day cycle; SD, short-day cycle; HT, high temperature; LT, low temperature; S/F, sterile/fertile plants; 5/6/7, 5th/6th/7th-stage; LOX, lipoxygenases; AOS, allene oxide synthase; AOC, allene oxide cyclase; OPR, oxophytodeinoate reductase.

To investigate the changes in JA during pollen development, we measured the activity levels of key enzymes, including LOX, AOS, AOC and OPR, that are related to the JA synthetic pathway in young spikelets and sword leaves sampled during different stages of development in the two lines induced by the photoperiod or temperature. Between the two PGMS rice types, the activities of the four JA synthetic enzymes in the tissues of D52S were generally higher than those in the tissues of NK58S (Figure 2 and Figure S3). This confirmed that the baseline JA contents in young spikelets and sword leaves were variety-specific, as mentioned above. The dynamic changes in enzyme activities showed that the change trends of LOX and AOS activities were obviously consistent in the same rice tissue not only during the different pollen developmental processes, but also between the two PGMS lines.

D52S and NK58S had different critical day lengths required for pollen development and exhibited opposite fertility outcomes under the same photoperiod. The tissue-specific changes in JA content and fertility indicators in combination with correlation analysis revealed that JA might be a link among the pollen fertility response, the photoperiod and the variety through the tissue- and period-specific regulation of its synthesis. Two enzymes, AOS and OPR, were downregulated in spikelets by the LD photoperiod in the 5th–6th stages; their activity was correlated with the LD photoperiod, with correlation coefficients of −0.54 to −0.65 (Figure S4). LOX was upregulated in spikelets in the 6th stage, and the JA content was upregulated in the 7th stage, with correlation coefficients of 0.63–0.73 (Figure S4). The indicators of fertility identified in this study included three 5th-stage spikelet enzymes, AOS, AOC and OPR (correlation coefficients of 0.59, −0.51 and 0.77, Figure S4); one 6th-stage leaf enzyme, AOS (correlation coefficients of 0.51, Figure S4); and the 7th-stage leaf JA and spikelet OPR (correlation coefficients of −0.59 and 0.56, Figure S4). Notably, the 5th-stage leaf JA content was most closely correlated with the 6th-stage spikelet JA content, while the 5th-stage spikelet JA content induced the upregulation of the 6th-stage leaf LOX and AOS enzyme activities (correlation coefficients of 1, Figure S4). Hence, enzyme activity is closely related to the temporal specificity and inter-tissue transfer of JA contents/synthesis in the early stage of pollen development.

For the PTGMS rice, PA64S, the correlation analysis revealed that the spikelet JA content was downregulated at LT, leading to pollen fertility recovery (Figure S5). In the process of JA synthesis, the enzyme activity levels of LOX and AOS showed a positive correlation with the JA content (correlation coefficients of 0.63–0.66 in the spikelet and 0.56–0.58 in the leaf, Figure S5). Young spikelets are highly influenced by different temperatures, which can lead to pollen fertility reversal. Interestingly, there was a negative correlation between the enzyme activity levels of AOC and OPR in the JA synthetic process in young spikelets (correlation coefficient of −0.63, Figure S5). Notably, AOC activities in the spikelets and leaves were significantly negatively correlated (correlation coefficient of −0.97, Figure S5). Moreover, there was also a significant negative correlation between the AOC enzyme activity and JA content in leaves (correlation coefficient of −0.83, Figure S5).

### 2.4. Analysis of JA Synthesis Gene Expression in Sterile and Fertile Lines by Transcriptome and qPCR Validation

To further elucidate the molecular basis of male sterility regulation, transcriptome analyses were performed, using the sterile- and fertile-line florets at the pistil and stamen formation stage (the 4th stage) of the spikelets. For each type of P(T)GMS rice of PA64S, D52S and NK58S, the significant differentially expressed genes (DEGs) between their sterile and fertile lines were selected with the criteria of *p*-value < 0.05 and fold change (FC) ≥ 2 (Figure S6A and Table S1). Compared with the D52S-S florets, a total of 1036 DEGs (3.49%), with 451 upregulated and 585 downregulated genes, were identified in the D52S-F florets. In NK58S-F, a total of 140 DEGs (0.46%), with 101 upregulated and 39 downregulated genes, were identified in the florets, compared with NK58S-S. For PA64S-F and PA64S-S, a total of 670 DEGs (2.12%), with 570 upregulated and 100 downregulated genes, were identified in the florets. Among these DEGs, twenty-seven genes were identified and shared in the three different types of P(T)GMS rice (Figure S6B). For example, there were four genes, Os01g0840100, Os01g0184100, Os03g0745000 and Os08g0500700, identified for encoding heat shock proteins; three ones, Os01g0550300, Os03g0241900 and Os07g0661400, encoding senescence-/dehydration-associated proteins; and also, one gene of Os08g0452500, encoding auxin responsive protein.

**Figure 3:**
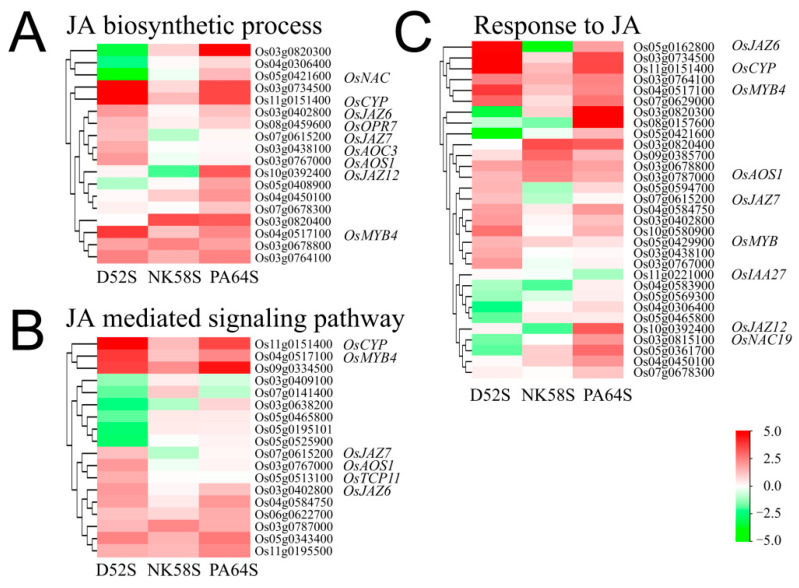
Heatmap analysis of gene expressions in JA biosynthetic process (**A**), JA signaling pathway (**B**) and response to JA (**C**) in 4th-stage-florets of PA64S, D52S and NK58S. Data are normalized and transformed by log_2_FC of gene expression in fertile plants, compared to those in sterile plants (*n* = 3). FC, fold change; S/F, sterile/fertile plants.

GO analysis suggested that the DEGs between the sterile and fertile lines were significantly enriched in common terms involved in JA, auxin and GA responses and the JA biosynthetic process (Table S2). Among those specifically involved in the JA biosynthetic process, the JA-mediated signaling pathway and the response to JA, forty-three genes participated in fertility regulation, most of which were upregulated in the 4th stage under LT- and LD-/SD conditions to restore all or partial fertility. Nearly all of these were significantly upregulated in PA64S-F, compared to PA64S-S (Figure 3). For example, of the upregulated JA biosynthetic genes with an FC of 5, set as the threshold for comparison between the fertile and sterile lines, there were five genes: Os03g0820300, Os11g0151400 (*cytochrome P450 94A1*), Os03g0734500, Os10g0392400 (*JAZ12*) and Os03g0820400 (*ZFP15*). These had FCs of 28.6, 8.4, 8.4, 6.9 and 6.8 in PA64S-F, one gene, Os03g0820400 (*ZFP15*), had an FC of 8.0 in NK58S-F, and three genes, Os11g0151400 (*cytochrome P450 94A1*), Os03g0734500 and Os04g0517100 (*MYB4*), had FCs of 39.9, 21.3 and 9.9 in D52S-F. Accordingly, we hypothesized that certain genes were involved in JA biosynthesis and rice pollen development and were regulated by the photoperiod and temperature.

**Figure 4:**
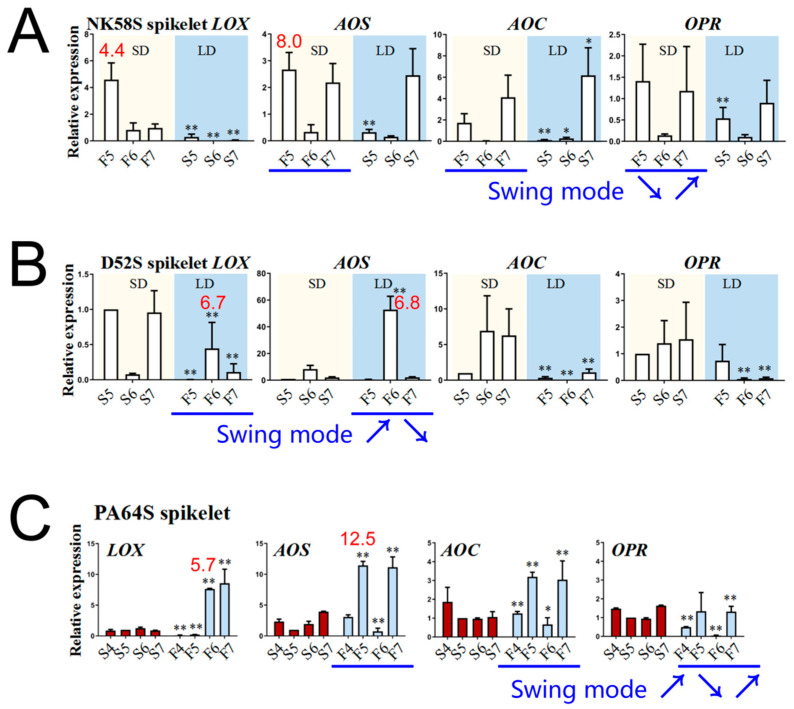
Expression analysis of JA synthesis enzyme genes in fertility-induced plants under different photoperiod and temperature conditions. The qPCR analysis of *LOX*, *AOS*, *AOC* and *OPR* genes in spikelets of NK58S (**A**), D52S (**B**) and PA64S (**C**) sterile/fertile lines from the 4th to 7th stages. The internal reference gene is *OsActin*. PA64S treated by HT/LT are columnar with the red and blue color, respectively. NK58S and D52S treated by LD/SD photoperiod are marked with a blue and yellow background, respectively. Data are shown as mean ± standard deviation (*n* = 3). Asterisks indicate significant differences between sterile and fertile plants of the same period revealed by Student’s *t*-test (*, *p* < 0.05; **, *p* < 0.01). The significant FC of gene expression in fertile plants compared to those in sterile plants are listed above the columnars in red. The blue arrows represent the trend and direction of gene expression change in fertile plants. LD, long-day cycle; SD, short-day cycle; HT, high temperature; LT, low temperature; S/F, sterile/fertile plants; 4/5/6/7, 4th/5th/6th/7th-stage.

In light of these results, we searched specifically for three genes, *OsAOS1* (Os03g0767000), *OsAOC3* (Os03g0438100) and *OsOPR7* (Os08g0459600), that directly participate in the JA biosynthetic process and that were induced to upregulated expression. *OsLOX* (Os03g0700700) was also included in the analysis. Through the qPCR assay, the expression of these genes was detected in the spikelets (Figure 4) and sword leaves (Figure S7) of the NK58S, D52S and PA64S sterile/fertile lines during pollen development in the sensitive period for male sterility, the 5th–7th stages. As shown, the gene expression levels of the three types of P(T)GMS rice obviously fluctuated during the 5th–7th stages in spikelets, with the peak expression level in the 5th–6th stages (Figure 4). These results indicate that the expression patterns revealed by the qPCR analysis of these genes were similar to those detected by high-throughput sequencing, confirming that the sequencing results and our analysis were accurate. Notably, *OsAOS1* expression was upregulated 6.8–12.5-fold in the spikelets from the fertile lines, compared to those from the sterile lines in the 5th–6th stages (Figure 4), and *OsAOS1* expression was upregulated 3.0–35.5-fold in the sword leaves from the fertile lines in the 5th-stage (Figure S7). Similarly, *OsLOX* expression was upregulated 2.7–30.0-fold in the spikelets and sword leaves of the sterile lines in the 5th–6th stages (Figure 4 and Figure S7).

These PTGMS and PGMS lines exhibited similar pollen abortion performance and processes, but they were controlled by different main effect factors (photoperiod or temperature). On the other hand, the LD/SD-PGMS lines NK58S and D52S exhibited opposite pollen fertility patterns under the same photoperiod. This result indicates that during rice pollen development, common genes involved in hormone synthesis and responses to hormone and environmental factors, such as JA biosynthesis genes, are upregulated. We deduced that JA could play a pivotal role in rice pollen development.

### 2.5. Methyl Jasmonate/Salicylhydroxamic Acid Spraying Treatment for Fertility Reversal in PGMS Rice

To confirm that JA is involved in fertility regulation, the sterile and fertile PGMS and PTGMS plants were treated by spraying with MEJA or SHAM to achieve fertility reversal. For the PGMS rice lines D52S and NK58S, spray application was effective in changing the pollen fertility as expected, and but it had no effect on PA64S (data not shown). The PGMS line was treated to obtain the presumed sterile/fertile plants, according to the basic photoperiod approach mentioned above. After spraying MEJA, 19.01% and 64.07% pollen grain fertility was detected in the anthers of young NK58S and D52S panicles, respectively (Table 2 and Figure S8). On the other hand, fewer fertile pollen grains (less than 10% pollen fertility) were detected in the anthers after spraying SHAM (Table 2). Under certain photoperiod conditions for the prospective sterile line, partial pollen fertility could be induced in NK58S and D52S by the MEJA treatment, leading to fertility reversal. Meanwhile, pollen fertility could be changed from prospective fertility induced by a certain photoperiod to male sterility by the SHAM spraying treatment, i.e., fertility reversal.

These results indicated that JA did participate in the regulation of rice anther development and pollen fertility. Pollen fertility in the PGMS rice line was significantly upregulated by MEJA spraying, leading to partial fertility (NK58F-F-R and D52S-F-R) and the reversal of the expected natural sterile conditions. Under SHAM spraying, pollen fertility also reversed, leading to relative male sterility (NK58F-S-R and D52S-S-R).

**Figure 5:**
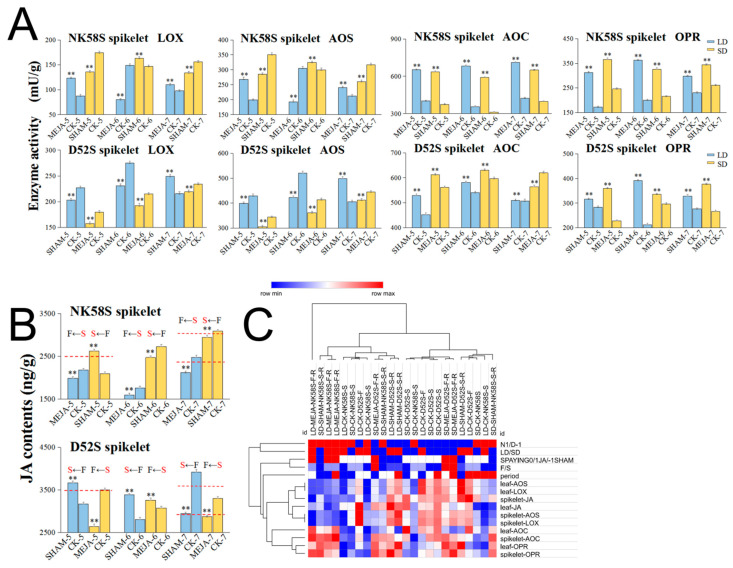
Analysis of JA synthesis enzyme activities and contents in D52S and NK58S by MEJA/SHAM-spraying treatment. The enzyme activities of LOX, AOS, AOC and OPR in the spikelets from NK58S and D52S (**A**), the JA contents (**B**), and heatmap analysis (**C**) are also listed. The LD/SD treated plants are columnar with blue/yellow color, respectively, and the spraying plants were named CK/MEJA/SHAM in the X-axis. The reversed-fertility plants are marked with arrows. The data homogenization for heatmap analysis are treated with maximum and minimum values of each indicator marked in red to blue. Data are shown as mean ± standard deviation (*n* = 3). Asterisks indicate significant differences of enzyme activities between spraying-induced reversed-fertility plants and those in the control group revealed by Student’s *t*-test (**, *p* < 0.01). LD, long-day cycle; SD, short-day cycle; MEJA, methyl jasmonate; SHAM, salicylhydroxamic acid; CK, control; S/F/R, sterile/fertile/fertility-reverse plants; 5/6/7, 5th/6th/7th-stage.

To verify the changes in JA contents and synthetic processes in response to spraying, we further measured the activities of the key enzymes LOX, AOS, AOC and OPR and the JA levels in young spikelets (Figure 5) and sword leaves (Figure S9) during different stages in the reversed-fertility plants induced by the spraying treatments. The results reflected that there were clear dynamic changes in the activities of the four key enzymes under the influence of the spraying treatments. Interestingly, AOC and OPR activities were upregulated in both young spikelets and sword leaves, regardless of which reagent was applied (Figure 5 and Figure S9). Notably, MEJA, SHAM and CK spraying were significantly correlated with pollen fertility, with a correlation coefficient of 0.66. This result demonstrated that MEJA and SHAM spraying was a key factor in inducing fertility reversal and had a greater impact on pollen development than environmental factors by regulating JA pathways. Furthermore, the leaf JA content had certain correlations with the spikelet JA content and the spikelet LOX and AOS activities (correlation coefficients of 0.47, 0.72 and 0.73). The effect of the spraying signal was transmitted between the tissues of young spikelets and sword leaves.

**Figure 6:**
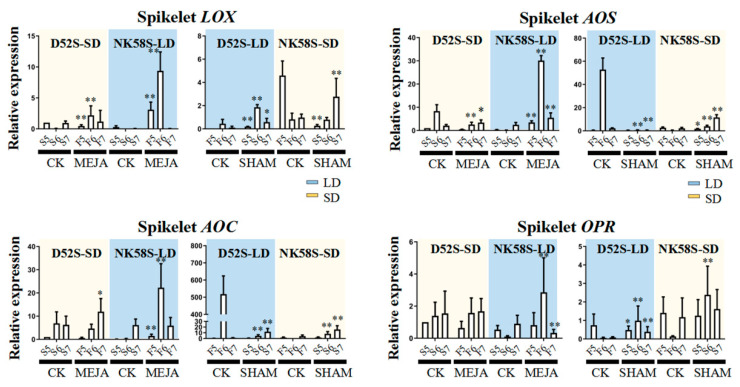
Expressions analysis of JA synthesis enzyme genes in fertility-reverse plants treated by MEJA/SHAM-spraying treatment. The qPCR analysis of *LOX*, *AOS*, *AOC* and *OPR* in the spikelets from NK58S and D52S sterile/fertile/fertility-reverse lines during 5th–7th-stage. The internal reference gene is *OsActin*. By photoperiod conditions and spraying treatment, the LD/SD-treated plants are marked with blue/yellow background, respectively. Data are shown as mean ± standard deviation (*n* = 3). Asterisks indicate significant differences of gene expression between spraying-induced reversed-fertility plants and those in the control group at the same period revealed by Student’s *t*-test (*, *p* < 0.05; **, *p* < 0.01). LD, long-day cycle; SD, short-day cycle; MEJA, methyl jasmonate; SHAM, salicylhydroxamic acid; CK, control; S/F/R, sterile/fertile/fertility-reverse plants; 5/6/7, 5th/6th/7th-stage.

Further, we searched specifically for the corresponding genes involved in the JA biosynthetic process, including *OsLOX,* *OsAOS1, OsAOC3* and *OsOPR7*, in the young spikelets and sword leaves of the plants in which fertility reversal was induced by the spraying treatments. As shown, the expression of these genes in NK58S and D52S leaves was obviously regulated by spraying, and the long-term response to spraying, particularly that in *OsAOS1*, *OsAOC3* and *OsOPR7* in leaves, was upregulated by MEJA spraying or downregulated by SHAM spraying (Figure S10). Notably, spikelet *OsAOS1* and *OsAOC3* expression during the 6th stage was upregulated 30–50-fold and 20–500-fold, respectively, in the fertile lines compared to the sterile lines by MEJA spraying and by photoperiod fertility induction (Figure 6).

## 3. Discussion

### 3.1. The Key Role of JA in Male Sterility in Rice

Hormones play important roles in plant growth and development and exhibit signal transmission and gene expression regulation functions at trace levels. Hormones are directly or indirectly involved in regulating pollen fertility during anther development, but this process is often the result of a combination of hormones and their balance. On the basis of transcriptome data from different P(T)GMS rice lines, we obtained data regarding a series of common hormone-related pathways and gene expression changes involved in pollen fertility regulation (Table S2 and Figure 3). Both the photoperiod and temperature significantly regulate the networks of various hormones, including JA, IAA and GA, in NK58S, D52S and PA64S (Figure 1). Through the determination of JA, IAA and GA levels, the changes in the hormone crosstalk and the effects of JA in the PGMS and PTGMS rice lines were characterized.

JA is widely distributed in plants and is derived from linolenic acid; it includes cyclopentanone group compounds, which can participate in the process of pollen development and plant defense and ageing [28,29,30]. The LD/SD-PGMS pair, NK58S and D52S, was treated with the same photoperiod during their sensitive fertility period but exhibited opposite fertility outcomes (Table 1 and Figure S1). The PTGMS PA64S used in the study exhibited relatively stable critical temperature characteristics related to fecundity for more than ten generations in directional temperature treatments [6]. The performance and processes of pollen abortion were similar in these PTGMS and PGMS lines but were controlled by different main factors, i.e., the photoperiod and temperature. All the lines showed typical male sterility (Table 1). On the basis of the transcriptome analysis of young spikes at the 4th stage, the expressions of JA synthesis-related genes were downregulated in sterile plants from these different types of P(T)GMS rice (Figure 3). These common JA biosynthesis genes were also verified to be upregulated during the rice pollen development response to fertility-inducing environmental factors. Thus, we deduced that JA signals and syntheses could play pivotal roles in rice pollen development, with a certain high dose of JA or JA biosynthesis upregulation in the initial phase of young spikelet differentiation, due to fertility-inducing environmental conditions.

We also tested and controlled the JA levels in the D52S and NK58S PGMS rice through exogenous MEJA and SHAM spraying and affirmed that JA plays a crucial role in the regulation of pollen fertility (Table 2 and Figure S8). Similar results were obtained in Arabidopsis *DAD1*, *OPR3* and *LOX3/4* double mutants and rice *OPR7* mutants with abnormal pollen maturation or eventually produced unmatched, defective anthers, which could restore fertility by treatment of exogenous JA/MEJA [23,40,41]. The results above indicate that JA is an important active hormone for pollen development and fertility recovery and that the regulation of JA synthesis is closely related to the mechanism of male sterility in plants. Moreover, a JA-mediated development delay exists in plants. In Arabidopsis, transcription factors affected senescence by activating the JA biosynthetic enzyme *LOX2* and then increasing JA levels [42]. In Arabidopsis mutant *AOS/LOX3/LOX4* treated with repetitive wounds, newly formed leaves were smaller and shortened, and the plants were often stunted [40,41]. The roles of JA in floral organs are also necessary for fertility, which has been identified in Arabidopsis and rice [23,43]. However, the MEJA and SHAM spraying had no effect on reversing pollen fertility for the PA64S PTGMS rice here. We speculate that the existence of multiple phytohormone crosstalk affects JA function and pollen fertility. For example, in cotton, a number of different *cis*-elements of *GhLOX* were identified in response to JA and GA signaling [44].

### 3.2. GA Specifically Regulates PA64S Pollen Fertility

In addition to JA, GA mediates normal pollen development in the early stages of the whole pollen development process. In this study, pollen fertility was upregulated by LT conditions, and the spikelet GA level was significantly upregulated in PA64S-F during the 5th–7th stages (Figure 1A). We previously studied male sterility in PA64S, which is significantly regulated by GA and the positive effector *GAMYB* and exhibits the same trends due to the delayed degradation of the tapetum [24]. The GRAS family is very important to GA signaling and regulates various aspects of plant growth and development [45]. *GRAS* (Os10g0551200) was found to respond to temperature changes to regulate male fertility in PA64S [46].

The SQUAMOSA-PROMOTER BINDING PROTEIN-LIKE (SPL) family of transcription factors is an important regulator of all stages of plant growth and development. The promotion of flowering by GA in Arabidopsis was demonstrated to involve an interaction between DELLA proteins and SPLs [47]. In Arabidopsis, miRNA156-*SPL* modules have been reported to regulate cell division and differentiation and to produce fertile pollen by regulating multiple *SPL*s [47,48]. In our previous study on male fertility in PA64S, a comprehensive analysis of miRNA and the degradome transcriptome showed that two key miRNA156-*SPL* pairs were found to respond to temperature changes to regulate male fertility [46].

**Figure 7:**
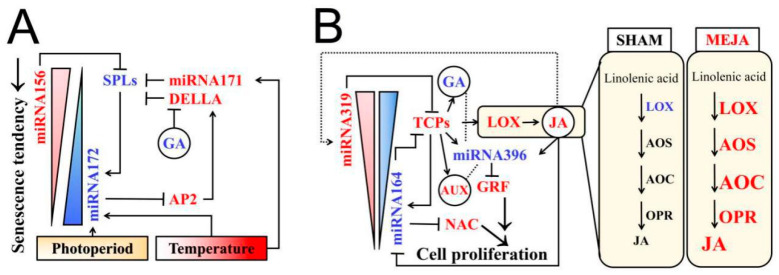
Interaction pattern between miRNA and phytohormone regulating pollen fertility in PTGMS and PGMS. (**A**) Work pattern of GA and miRNA156-miRNA172 in senescence mechanism. (**B**) Crosstalk pattern of JA, IAA, GA and miRNA164-miRNA319 in cell proliferation mechanism. Compared to those in sterile plants, miRNAs and their target genes downregulated/upregulated in fertile spikelets are marked in blue/red color according to our previous results of PA64S [46]. For spray treatment of PGMS, gene expressions of JA synthesis downregulated/upregulated in fertile NK58S and D52S spikelets are marked in blue/red color, according to data in Figure 5 and Figure 6.

Accordingly, GA may play a regulatory role in the relationship between DELLA and SPL by participating in the miRNA module to regulate PA64S *SPLs* (Figure 7A). GA is well known for its role in promoting floral transition through the degradation of the transcription repressor DELLA. It has been demonstrated that GA promotes flowering through miRNAs in Arabidopsis [47]. MiRNA156/172 is a highly conserved regulatory module that controls flowering competency and timing in plants [49]. The miRNA overexpression causes the exact opposite effect of the vegetative-phase transition, delaying or promoting flowering in Arabidopsis [50,51]. MiRNA156/172 regulation is tightly connected, and their expression levels are oppositely affected by developmental processes and the environmental factors temperature and light; that is, plant developmental transitions are coordinated by their antagonistic activities [52,53]. These studies briefly illustrate the complexity of GA and miRNA regulatory networks in controlling plant development.

The principle that stimulates photoperiod- and thermo-sensitive male sterility may contribute to inducing pollen fertility reversal through the miRNA module as well as the plant hormone signaling network. Current studies have reported that slow development underlies fertility recovery in Arabidopsis PGMS and TGMS lines; this allows plants to apply different strategies to overcome pollen formation defects, particularly in TGMS plant lines [24,54]. Cytological observations have also shown that PA64S pollen development slightly slows if low temperatures (below 23.5 °C) are induced for years (data not shown). Given that male reproductive development is conserved in plants, we propose that slowing development is a general mechanism for pollen fertility recovery that is applicable to sterility–fertility conversion in the PTGMS PA64S line induced by LT conditions; this conversion could also be controlled through GA-miRNAs regulatory networks (Figure 7A). However, we have only just begun to understand the molecular bases of this conversion beyond environmental factors, phytohormone signals and miRNA crosstalk. Additional research is needed to resolve how these components together regulate rice pollen fertility.

### 3.3. A Possible Model for miRNAs and Phytohormones (JA, GA) Crosstalk Network in Rice Fertility Regulation

Phytohormones, including auxin (AUX), GA and JA, control most developmental stages in plants. In multiple phytohormone pathways, miRNA is a regulator of mitotic cell division by regulating GROWTH RESPONDING FACTOR (GRF), TEOSINTEBRANCHED1/CYCLOIDEA/PROLIFERATING CELL FACTOR (TCP) and NAC domain-containing proteins and thus controlling or determining the cell fate of development and senescence [45,47,48,49,50,51,52,53,54,55,56,57,58,59]. In our previous research, miRNA396c-*GRF1*/*3*, *PCF6/7* and *NAC* were found to respond to temperature changes, thereby regulating PA64S male fertility [46]. A substantial number of genes have been found to be involved in GA and JA signaling pathways, including the secondary targets of miRNA396, *AtGA3ox1* and *AtGA20ox*, which are directly involved in GA biosynthesis [55,56], and the targets *JAZ3/7* and *JAR1*, which are involved in bioactive JA biosynthesis. Accordingly, a possible model for the phytohormones crosstalk network in rice fertility development due to the complexity of environmental factor-mediated regulation is established here (Figure 7B).

**Figure 8:**
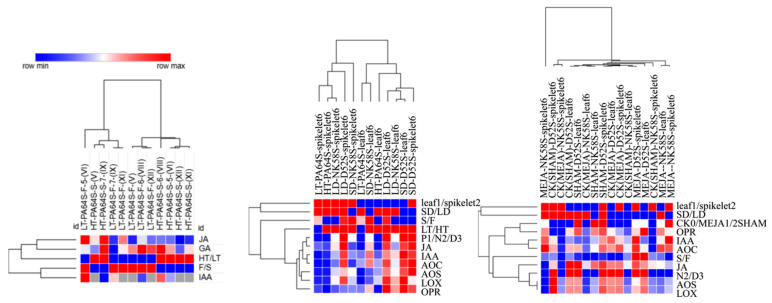
Heatmap analysis of hormone contents and JA synthesis enzyme activities in D52S, NK58S and PA64S. Data are from Figure 1, Figure 2 and Figure 5, and are treated for homogenization with maximum and minimum values of each indicator marked in red to blue. LD, long-day cycle; SD, short-day cycle; HT, high temperature; LT, low temperature; MEJA, methyl jasmonate; SHAM, salicylhydroxamic acid; CK, control; S/F/R, sterile/fertile/fertility-reverse plants; 5/6/7, 5th/6th/7th-stage.

AUX and JA play important roles in regulating plant sex differentiation, mediating stamen and pollen maturation, and controlling floral organ development [56,57]. In this study, both the photoperiod and temperature significantly regulated various JA and IAA hormone networks in the different PGMS and PTGMS lines, most obviously in the PGMS lines NK58S and D52S (Figure 8). Furthermore, the PGMS rice exhibited fertility reversal under the MEJA and SHAM spraying treatments (Table 2). The initial phase of young spike differentiation is generally a fertility induction phase that is devoid of cellular changes through the 3rd–4th stages. During this period, many DEGs related to hormone biosynthesis and responses to hormone processing were significantly enriched in all three of the PTGMS/PGMS rice lines. The effects of the fluctuation of hormones, including IAA, JA and GA, were highly dynamic during the 5th–6th stages in the three lines (Figure 8). In PA64S, we found that the identified miRNAs and their target genes had stronger expression trends in the 6th stage, which was more suitable for the exhibition of differences in miRNA to regulate fertility [46]. Hormone functions may involve crosstalk with miRNA signals, thereby reflecting the plant developmental status. MiRNAs related to male sterility in plants have received more attention during the past decade than in previous decades. The roles of certain important miRNAs identified in our earlier work to communicate with GA and JA are clarified in this study (Figure 7). Taken together, these data suggest that the module might be an important channel through which both auxin and JA control cell proliferation in response to abiotic stresses.

The overall control of plant development by auxin involves various TCP, NAC and GRF transcription factors and is further linked to JA and GA. In the PTGMS and PGMS rice lines, the genes involved in the JA biosynthetic process and the responses to JA were upregulated by the LD/SD photoperiods and LT conditions (Figure 3), which completely or partially restored their fertility (Table 1). Thus, we deduced that JA is a key component of rice pollen development and that a certain high dose of JA upregulates the initial phase of young spike differentiation and subsequently sustains its activity with regard to its signaling function. Through exogenous MEJA and SHAM spraying, JA was also shown to reverse PGMS pollen fertility (Table 2). The functions of JA seem to involve environmental factors and crosstalk with other hormone signals through miRNAs; and our study provides a JA-oriented perspective on the network.

## 4. Materials and Methods

### 4.1. Plant Materials

The genic male-sterile rice materials PA64S, D52S and NK58S were identified and bred for multiple generations by our research team [24,58]. PA64S is a PTGMS line derived from a cross between NK58S (*Oryza sativa* L. ssp. *japonica*) × Peiai 64 (*Oryza sativa* L. ssp. *indica*). PA64S is affected by the photoperiod but exhibits mainly temperature-sensitive characteristics, and the critical temperature for male sterility in PA64S is 23.5 °C [6]. Under LD conditions, male sterility manifested when the average temperature was above 23.5 °C, and males were fertile at average temperatures below 23.5 °C.

A LD- and SD-sensitive pair of PGMS lines, NK58S and D52S, respectively, was also used in this study. D52S is a SD-PGMS line that manifests male sterility under SD conditions and is derived from a cross between XanB3 (*Oryza sativa* L. ssp. *indica*) × Hongjin (*Oryza sativa* L. ssp. *japonica*). The two-line system of D52S is fertile under LD but sterile under SD. In contrast, NK58S is a LD-PGMS rice that is fertile under SD conditions but sterile under LD conditions.

### 4.2. Rice Planting and Treatment under Sterile/Fertile Photoperiod and Temperature Conditions

The experiment was conducted at the Crop Physiology and Production Centre of Huazhong Agricultural University (30°28′ N, 114°20′ E). The experiment was carried out from 2017 to 2020 with rice seeds from our laboratory. On 10 May of each year, seedlings showing good growth were transplanted into enamel pots with a height of 20 cm and a diameter of 16 cm and grown under natural light and temperature conditions. From May to September during the growing season, three plants were grown in each pot and were restricted to producing only primary tillers. Regular fertilization, watering, and disease management practices were carried out.

For PA64S, when 50% of the plant population had reached the secondary stalk and spikelet primordium differentiation stage (the 3rd stage) according to the eight-stage differentiation of young spikelets [39], the plants were treated with high and low temperatures (HT, 28 °C; LT, 21 °C) in a manually controlled plant growth chamber HP1500GS-B (Ruihua, Wuhan, China). The temperature ranges were set according to those established our previous studies [24,46]. In addition, the growing environment conditions were set with fluorescent lighting at 300 μmol·m^−2^·s^−1^, LD conditions (a 14 h light and 10 h dark cycle) and a relative humidity of 80%. The temperature treatment was sustained until the pollen-filling stage (the 7th stage). Then, all treated plants were grown under natural conditions until maturity. Accordingly, sterile and fertile plants (PA64S-S and PA64S-F) were obtained under HT and LT, respectively.

For NK58S and D52S, the plants were also subjected to the treatments in a manually controlled growth chamber during the sensitive period mentioned above (the 3rd to 7th stages). The conditions were set to a LD cycle of 14 h light at 28 °C and 10 h dark at 26 °C and a SD cycle of 10 h light at 28°C and 14 h dark at 26 °C; the fluorescent lighting and relative humidity conditions were the same as those described in our previous studies [58]. Accordingly, sterile/fertile NK58S plants (NK58S-S/NK58S-F) were obtained under LD/SD conditions (14 h day/10 h day). The sterile/fertile D52S plants were obtained under the opposite conditions; that is, D52S-S and D52S-F were obtained under SD and LD cycles, respectively. The treatment groups were randomized according to all sources of variation in the treatments, i.e., the exact temperature, humidity and light conditions.

### 4.3. MEJA and SHAM Spraying Treatment for Fertility Reversal

In addition to the temperature and photoperiod treatments, when 50% of the plant populations of PA64S, D52S and NK58S had reached the initial sensitive period (the 3rd stage) mentioned above, the plants were treated by spraying a MEJA or salicylhydroxamic acid (SHAM) solution to reverse their fertility. The MEJA and SHAM solutions were prepared with 1 mM MEJA or 0.5 mM SHAM solution, respectively, dissolved into a base solution of 0.2% dimethyl sulfoxide as the cosolvent and 0.1% Tween-20 as the surfactant. A spraying treatment with only the base solution was also carried out as a control (CK). Spraying was performed at 6:00 p.m. only once, at the 3rd stage. The standard treatment was to wet each plant immediately until the solution began to drip off the leaves [59]. The MEJA solution was sprayed on the presumed-sterile plants, and the SHAM solution was sprayed on the presumed-fertile plants. The fertility status of the plants was predicted on the basis of the temperature and photoperiod treatments described above.

### 4.4. Phenotype Identification and Characterization and Sampling of Sterile and Fertile Plants

To observe the morphological differences between mature sterile and fertile plants, the anthers of the top floret in which heading had initiated were stained with a 1% potassium iodide solution, as previously described [46]. The anthers were observed in at least three fields viewed randomly by an Olympus model BH2 microscope (Olympus, Tokyo, Japan). Fertile and abortive pollen was detected, and the pollen fertility percentage was calculated and averaged. At least 60 plants with more than two young spikelets per plant were sampled for this study (*n* ≥ 120). Significant differences were determined, according to Student’s *t*-test. Accordingly, sterile and fertile plants (cultivar-S/F) were obtained and confirmed under temperature and photoperiod treatments (LD/SD cycles and HT/LT conditions), including the reversed-fertility plants subjected to the MEJA/SHAM spraying treatments.

According to the eight-stage differentiation process for young spikes mentioned above, we sampled the florets of plants that were in the pistil and stamen formation stage (the 4th stage), the pollen mother cell formation and meiosis stages (the 5th and 6th stages), and the pollen-filling stage (the 7th stage). We selected 60 plants with more than two young spikelets per plant from each treatment and then immediately froze them in liquid nitrogen for physiological and biochemical determination and molecular validation.

### 4.5. RNA Extraction and RNA Sequencing

For PA64S, D52S and NK58S under the temperature and photoperiod treatments, the total RNA was extracted from florets of the sterile and fertile plants of each line in the pistil and stamen formation stage (the 4th stage), using an RNAprep Pure Plant Kit (TIANGEN, Beijing, China) as described by the supplier. Total RNA was inspected RNA integrity by an Agilent Bioanalyzer 2100 (Agilent technologies, Santa Clara, CA, USA) with RNA Integrity Number (RIN) > 7.0 and further purified by RNeasy micro kit (QIAGEN, Hilden, Germany).

For RNA sequencing, 3 µg of RNA from each sample was used. RNA-seq and bioinformatics analyses were conducted by Novogene (Beijing, China). The reference genome information was obtained from the Ensemble database of *Oryza sativa* L. *japonica* (http://ensemblgenomes.org/, accessed on 20 May 2020). Sequencing libraries were generated by a cBot Clonal Amplification System (Illumina, San Diego, CA, USA) and clustered on a cBot Cluster Generation System using TruSeq SR Cluster Kit v3-cBot-HS (Illumina, San Diego, CA, USA) according to the manufacturer’s instructions. After cluster generation, the library preparations were sequenced on an Illumina HiSeq 2500 platform (Illumina, San Diego, CA, USA). Each sample was represented by three biological replicates.

For mapping the genome, clean reads obtained by removing reads containing adapters were mapped to the reference genome (MSU *Oryza* v7.0, http://rice.plantbiology.msu.edu/annotation_pseudo_current.shtml, accessed on 17 June 2021) using Hisat2 (https://daehwankimlab.github.io/hisat2/, accessed on 17 June 2021). The mapped reads were assembled by StringTie (http://ccb.jhu.edu/software/stringtie/index.shtml, accessed on 17 June 2021). FeatureCounts v1.5.0-p3 (WEHI, Parkville Victoria, Australia) was used to count the read numbers mapped to each gene. Then, the fragments per kilobase per million (FPKM) of each gene were calculated based on the length and read count mapped by Cufflinks (http://cufflinks.cbcb.umd.edu, accessed on 17 June 2021) [60]. The differential gene expression analysis was performed using the DESeq2 R package and Cufflinks. A corrected *p*-value of 0.05 and absolute fold change (FC) of 2 were set as the thresholds for significant differential expression. Gene ontology (GO) enrichment analysis of the differentially expressed genes (DEGs) was implemented by the clusterProfiler R package.

### 4.6. Quantitative Real-Time PCR Validation of Gene Expression

To confirm the expression of genes involved in the JA biosynthetic process, we selected a total of four genes for quantitative real-time PCR (qPCR) verification, including *OsLOX* (Os03g0700700), *OsAOS1* (Os03g0767000), *OsAOC3* (Os03g0438100) and *OsOPR7* (Os08g0459600). The primers for the candidate genes designed by NCBI were synthesized by Shenggong (Bioengineering Co., Ltd., Shanghai, China, Table S3), and *Actin7* (X16280) was used as an internal reference gene [46].

For PA64S, D52S and NK58S under the temperature and photoperiod treatments and the MEJA/SHAM spraying treatments, the total RNA was extracted from the spikelets and sword leaves of the sterile and fertile plants of each line in the 4th–7th stages, using the RNAprep Pure Plant Kit (TIANGEN, Beijing, China). First-strand cDNA was synthesized in a 20-µL reaction solution with oligo(dT) primers, using a ReverTra kit (TOYOBO, Osaka, Japan). qPCR was performed in a 10-μL volume using SYBR Green SuperMix (Bio-Rad, Hercules, CA, USA) on a QuantStudio 6 Flex instrument (Applied Biosystems, Foster, CA, USA). With three biological replicates, the relative gene expression was analyzed by the relative quantitative method [61].

### 4.7. Measurement of LOX, AOS, AOC and OPR Enzyme Activities and Plant Hormone Contents

For PA64S, D52S and NK58S, the activities of key enzymes in the JA biosynthetic process were analyzed in the spikelets and sword leaves of the sterile and fertile plants of each line in the 5th–7th stages under the temperature, photoperiod and spraying treatments.

The activities of four key enzymes, including LOX, AOS, AOC and OPR, were determined by the double antibody sandwich method [62]. Purified antibodies against LOX, AOS, AOC and OPR were used to coat the microplate to generate solid-phase antibodies (Jiangsu KeJing Biological Technology Co., Ltd., Yancheng, China). First, a crude enzyme sample was extracted with a cold extraction buffer (pH 8.2) comprising 50 mM HEPES–KOH. After 20 min of chilling, the mixture was centrifuged at 8000× *g* for 15 min at 4 °C. The supernatant was then added to the micropores of the coated monoclonal antibody. Then, the micropores were assayed at a wavelength of 450 nm, using a fluorescence microplate reader (Infinite 200 NanoQuant, Tecan, Mannedorf, Switzerland) combined with horseradish peroxidase-conjugated reagent, with three independent replicates for each group. The measured protein concentrations were converted into enzyme activity (mU/g, FW) using empirical coefficients.

Simultaneously, the plant hormone contents of the florets of the sterile and fertile plants of each line were analyzed at the 5th, 6th and 7th stages under the different temperature, photoperiod and spraying treatments. The JA, indole-3-acetic acid (IAA), and gibberellin (GA) contents were determined by the double antibody sandwich method mentioned above, using purified antibodies against JA, IAA and GA (Jiangsu KeJing Biological Technology Co., Ltd., Yancheng, China) [62]. Each sample was assayed with three independent replicates. The JA, IAA and GA concentrations measured were converted into fresh tissue contents (ng/g, FW).

### 4.8. Data Analysis

The data were normalized to the total peak area of each sample and transformed by log2 in Excel software (Microsoft Corp., Redmond, WA, USA). Statistical analyses of the hormone contents and the activity values were conducted by analyses of variance and significant differences, using Excel software. The correlation of the trend between variables was analyzed according to the Pearson correlation coefficient using an online platform (http://www.bioinformatics.com.cn, accessed on 17 June 2021). A heatmap was generated with an online platform (https://software.broadinstitute.org/morpheus/, accessed on 17 June 2021) and platform-independent software (https://github.com/CJ-Chen/TBtools/releases, accessed on 17 June 2021).

## 5. Conclusions

Based on the transcriptomes of fertile and sterile plants of P(T)GMS rice lines, we propose that the JA level and the JA biosynthetic pathway are pivotal for viable pollen production. The JA signaling mode involves initial upregulation, constant fluctuation and leaf-spikelet signaling under certain fertility-inducing conditions. Through biochemical and molecular validation, the activities of two key enzymes, AOS and AOC, in response to MEJA and SHAM spraying were identified in PGMS. The spraying treatments effectively reversed pollen fertility by influencing the JA biosynthetic pathway. The results of this study provide a new understanding of the hormone interactions and networks that regulate pollen fertility in male-sterile rice.

## Figures and Tables

**Table 1:** Pollen fertility of PA64S, D52S and NK58S by photoperiod and temperature treatment.

Photoperiod and Temperature Condition	S/F Plant	Pollen Fertility (%)
LD-NT (about 27 °C)	NK58S-S	4.63 ± 1.87 **
SD-NT (about 27 °C)	NK58S-F	70.61 ± 5.49
SD-NT (about 27 °C)	D52S-S	1.34 ± 0.30 **
LD-NT (about 27 °C)	D52S-F	48.93 ± 2.01
LD-HT (28 °C)	PA64S-S	0.00 ± 0.00 **
LD-LT (21 °C)	PA64S-F	41.35 ± 1.76

Values are expressed as mean ± standard deviation, *n* = 120. ** Asterisks indicate significant differences between S and F plants revealed by Student’s *t*-test (*p* < 0.01). LD, long-day cycle of 14 h-light and 10 h-dark; SD, short-day cycle of 10 h-light and 14 h-dark; HT, high temperature; NT, normal temperature, LT, low temperature; S/F, sterile/fertile plants.

**Table 2:** Pollen fertility of D52S and NK58S by a MEJA/SHAM-spraying treatment.

Photoperiod Condition	D52S			NK58S		
	Spraying	S/F Plant	Pollen Fertility (%)	Spraying	S/F Plant	Pollen Fertility (%)
LD	Control	D52S-F	21.54 ± 4.91	Control	NK58S-S	0.25 ± 0.01
	0.5 mM SHAM	D52S-S-R	8.63 ± 1.78 **	1 mM MEJA	NK58S-F-R	19.01 ± 2.66 **
SD	Control	D52S-S	0.54 ± 0.01	Control	NK58S-F	21.54 ± 6.11
	1 mM MEJA	D52S-F-R	64.07 ± 7.00 **	0.5 mM SHAM	NK58S-S-R	0.19 ± 0.01 **

Values are expressed as mean ± standard deviation, *n* = 120. ** Asterisks indicate significant differences of pollen fertility between spraying-induced reversed-fertility plants and those in the control group revealed by Student’s *t*-test (*p* < 0.01). LD, long-day cycle of 14 h-light and 10 h-dark; SD, short-day cycle of 10 h-light and 14 h-dark; S/F/R, sterile/fertile/fertility-reverse plants; MEJA, methyl jasmonate; SHAM, salicylhydroxamic acid.

## Supplementary Materials

The following are available online at https://www.mdpi.com/article/10.3390/ijms22157926/s1.
