# Supplementary material for: Jasmonic Acid Plays a Pivotal Role in Pollen Development and Fertility Regulation in Different Types of P(T)GMS Rice Lines
Source: Int J Mol Sci. 2021 Jul 25;22(15):7926. doi: 10.3390/ijms22157926 (PMC8348444; doi:10.3390/ijms22157926)
Supplement: Supplementary file 1 [file ijms-22-07926-s001.zip › ijms-1256250-supplementary.pdf]

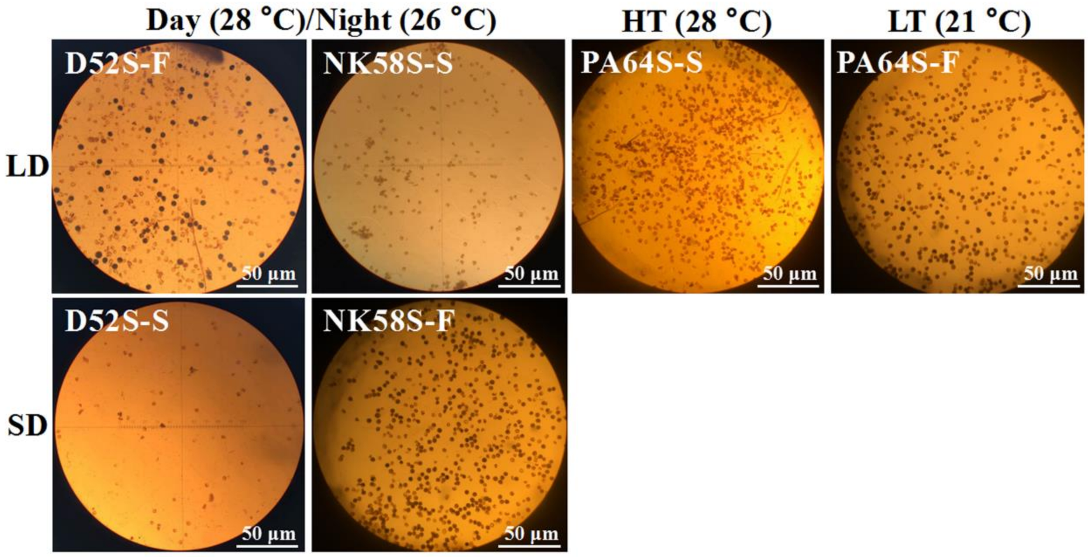

Figure S1. Pollen staining of PA64S, D52S and NK58S under photoperiod and temperature treatment. LD, long-day cycle of 14 h-light and 10 h-dark; SD, short-day cycle of 10 h-light and 14 h-dark; HT, high temperature; LT, low temperature; S/F, sterile/fertile plants.

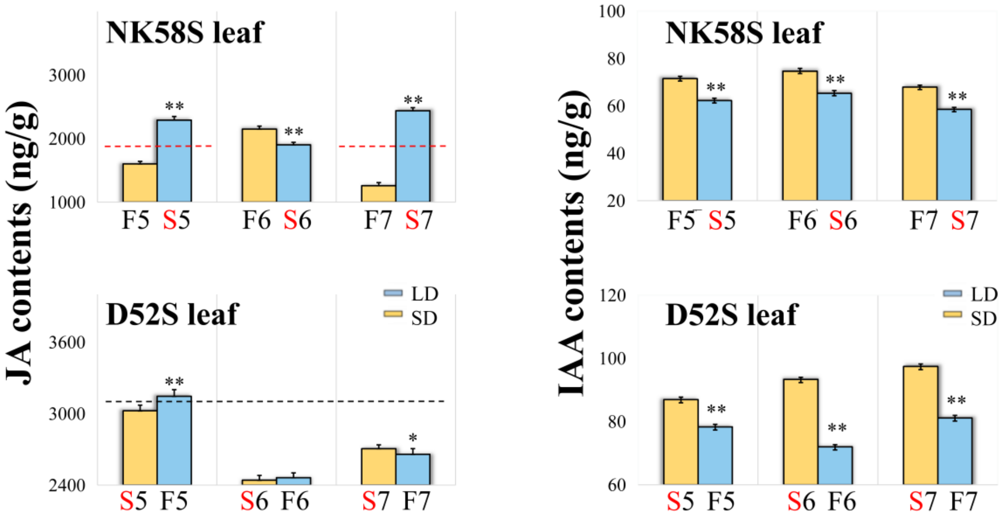

Figure S2. Analysis of JA and IAA contents of sword leaf in D52S and NK58S by photoperiod treatment. The contents from LD/SD-treated plants are columnar with blue/yellow colour, respectively. Data are shown as mean ± standard deviation (n = 3). Asterisks indicate significant differences between fertile and sterile plants revealed by Student's *t*-test (*, $P < 0.05$; **, $P < 0.01$) in the same period. LD, long-day cycle; SD, short-day cycle; S/F, sterile/fertile plants; 5/6/7, $5^{th}/6^{th}/7^{th}$-stage; JA, jasmonic acid; IAA, indole-3-acetic acid.

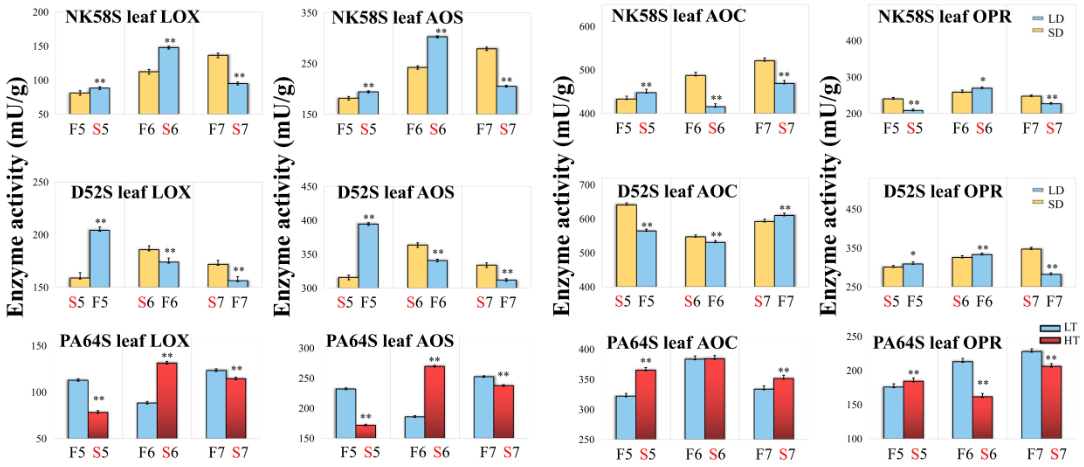

Figure S3. Four key enzyme activities of JA synthesis pathway in D52S, NK58S and PA64S leaves by photoperiod and temperature treatment. The LD/SD and HT/LT treated plants are columnar with blue/yellow and red/blue colour, respectively. Data are shown as mean ± standard deviation (n = 3). Asterisks indicate significant differences between fertile and sterile plants revealed by Student's *t*-test (*, $P < 0.05$; **, $P < 0.01$). LD, long-day cycle; SD, short-day cycle; HT, high temperature; LT, low temperature; S/F, sterile/fertile plants; 5/6/7, $5^{th}/6^{th}/7^{th}$-stage; LOX, lipoxygenases; AOS, allene oxide synthase; AOC, allene oxide cyclase; OPR, oxophytodeinoate reductase.

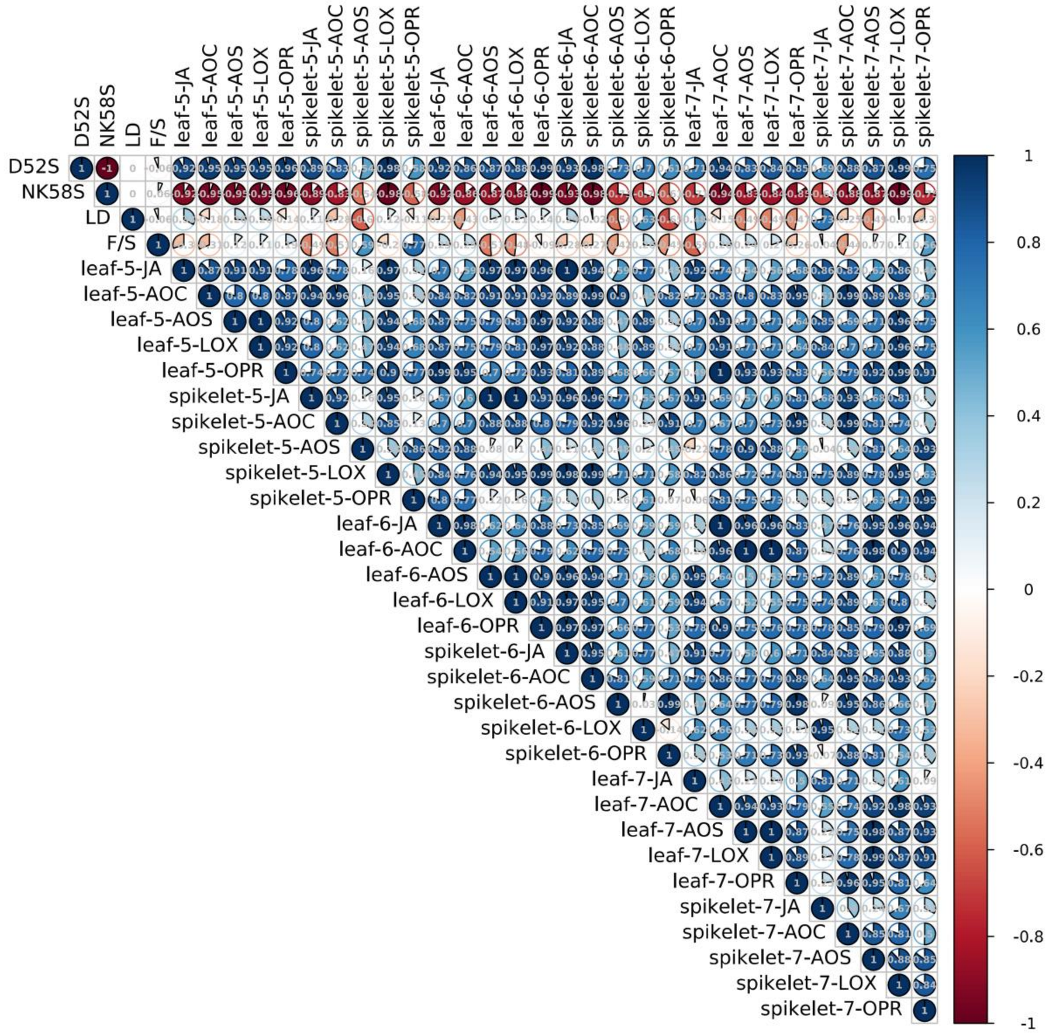

Figure S4. Heatmap of correlation analysis of enzyme activities and pollen fertility rate in D52S and NK58S. Pearson correlation coefficient of indicators are listed, and data are from Figure 1 and 2, Figure S2 and S3.

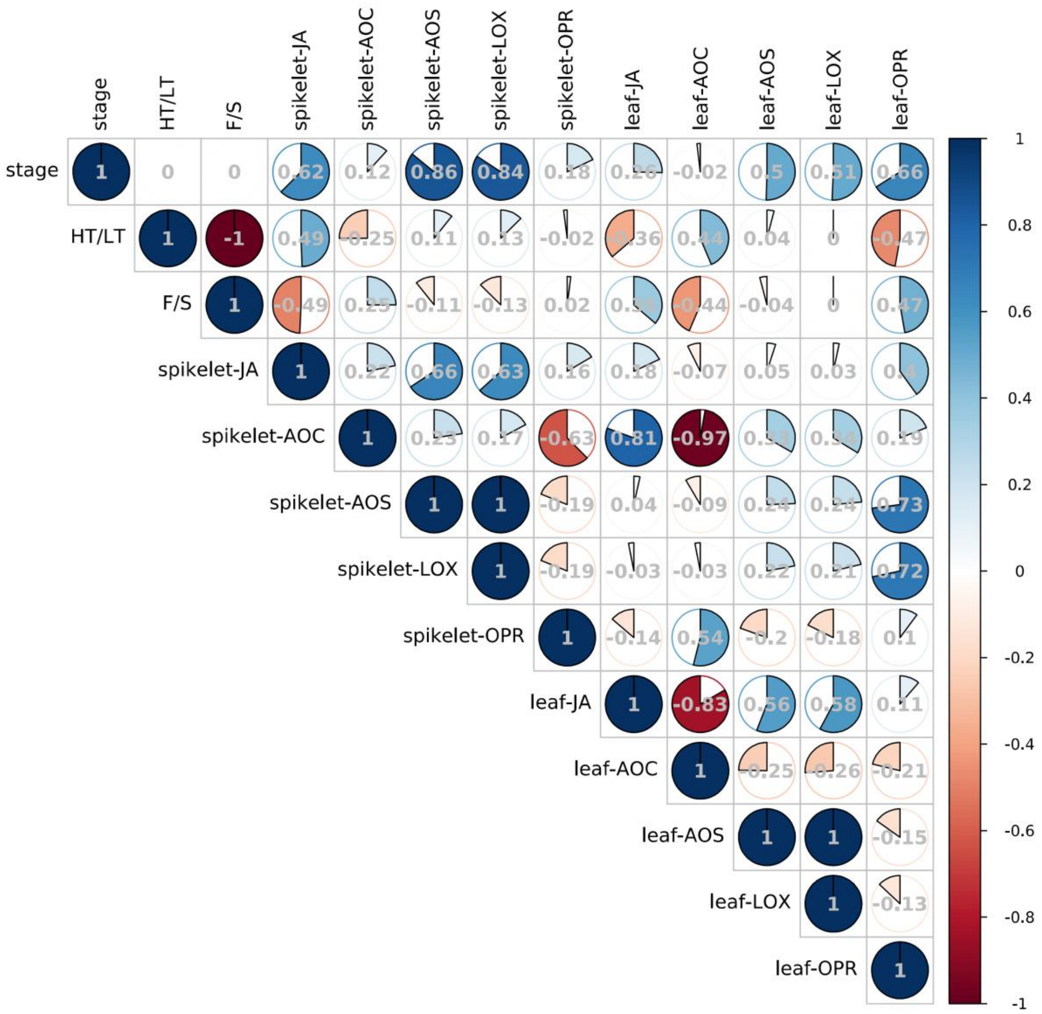

Figure S5. Heatmap of correlation analysis of enzyme activities and pollen fertility rate in PA64S. Pearson correlation coefficient of indicators are listed, and data are from Figure 1 and 2, Figure S2 and S3.

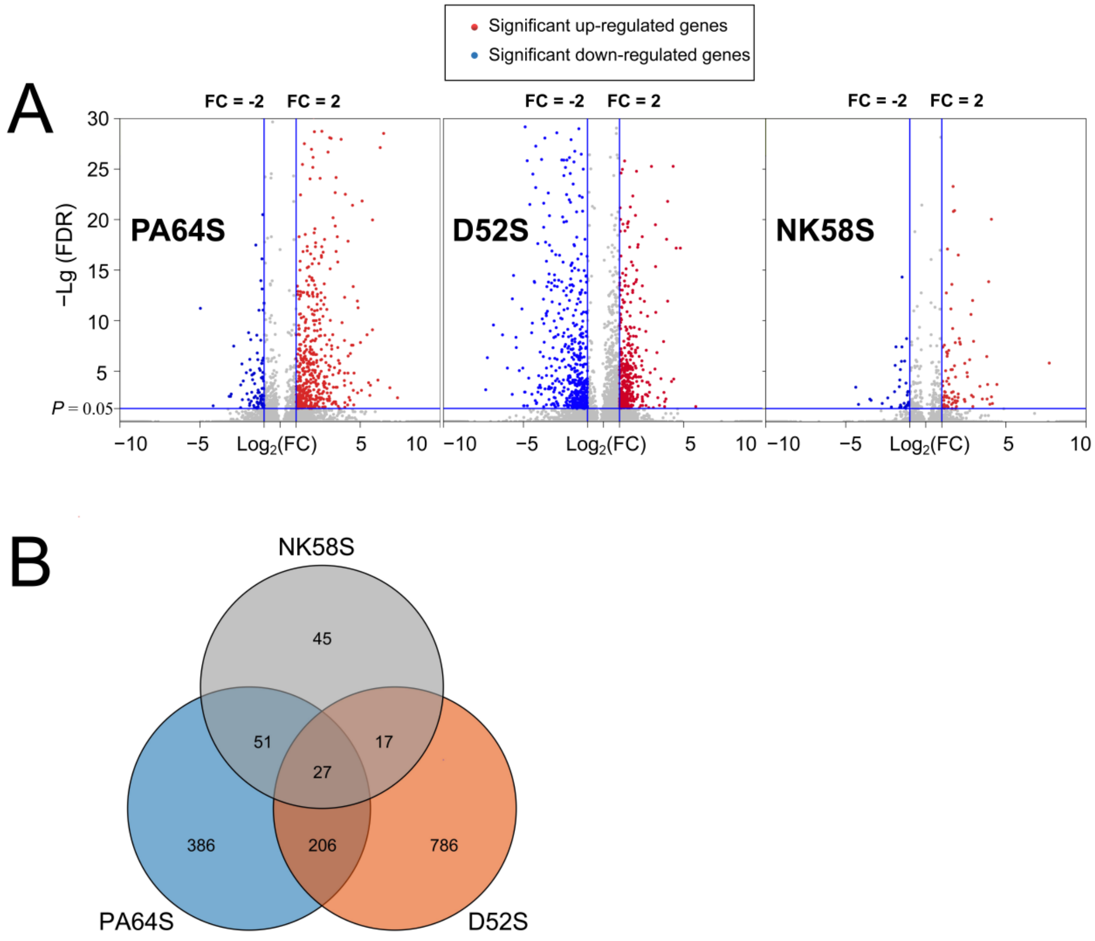

Figure S6. Analysis of DEGs between fertile and sterile spikelets from different types of P(T)GMS rice lines. Volcanic maps (A) show DEGs in the 4th-stage spikelets between fertile and sterile plants from PA64S, D52S and NK58S. The X-axis indicates the DEGs expression levels with the value of log_2(FC), of which FC compares the ratio of FPKM of DEGs in fertile plant to those in sterile plant, and the Y-axis indicates the significance of DEGs differential expression with the value of -lg(FDR). Two vertical blue lines indicate FC = 2, and one horizontal blue line indicates P = 0.05. A Venn diagram (B) shows the unique and shared DEGs between fertile and sterile spikelets from three P(T)GMS lines. DEG, differentially expressed genes; FPKM, fragments per kilobase of exon model per million mapped reads; FC, fold change.

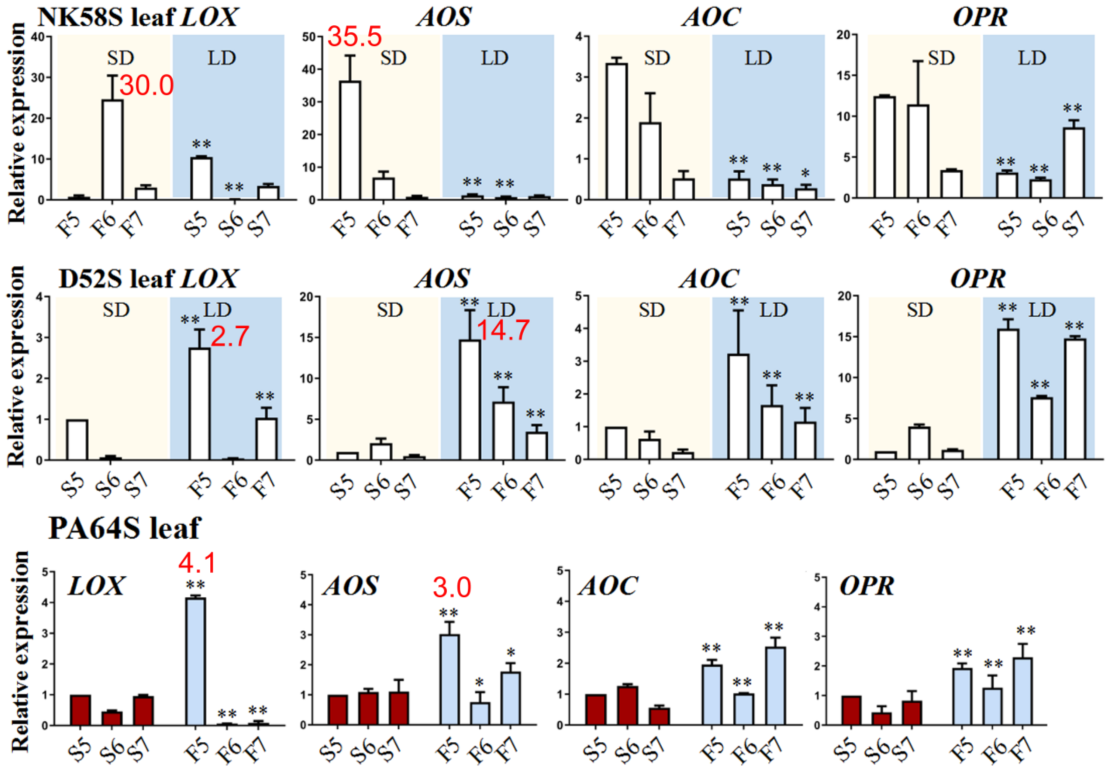

Figure S7. Expression analysis of JA synthesis enzyme genes in fertility-induced plants under different photoperiod and temperature conditions. The qPCR analysis of *LOX*, *AOS*, *AOC* and *OPR* genes in leaves of NK58S, D52S and PA64S sterile/fertile lines from 5th to 7th stage. The internal reference gene is *OsActin*. PA64S are treated by HT/LT are columnar with red and blue colour, respectively. NK58S and D52S treated by LD/SD photoperiod are marked with blue and yellow background, respectively. Data are shown as mean ± standard deviation (n = 3). Asterisks indicate significant differences between fertile and sterile plants revealed by Student's *t*-test (*, $P < 0.05$; **, $P < 0.01$). The significant FC of gene expression in fertile plants compared to those in sterile plants are listed above columnar in red. LD, long-day cycle; SD, short-day cycle; HT, high temperature; LT, low temperature; S/F, sterile/fertile plants; 5/6/7, 5th/6th/7th-stage.

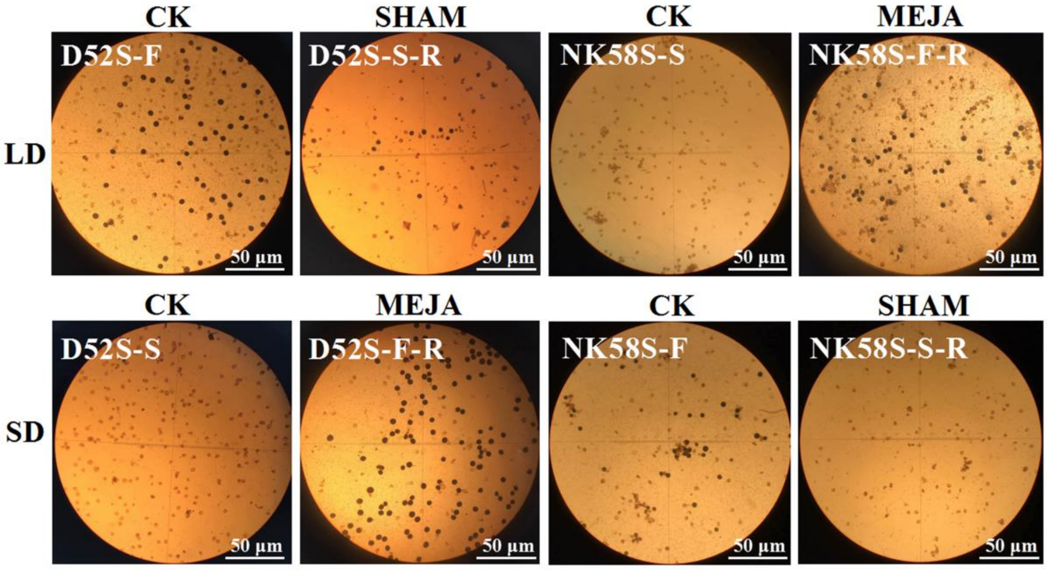

Figure S8. Pollen staining of D52S and NK58S by MEJA/SHAM-spraying treatment. LD, long-day cycle; SD, short-day cycle; MEJA, methyl jasmonate; SHAM, salicylhydroxamic acid; S/F/R, sterile/fertile/fertility-reverse plants.

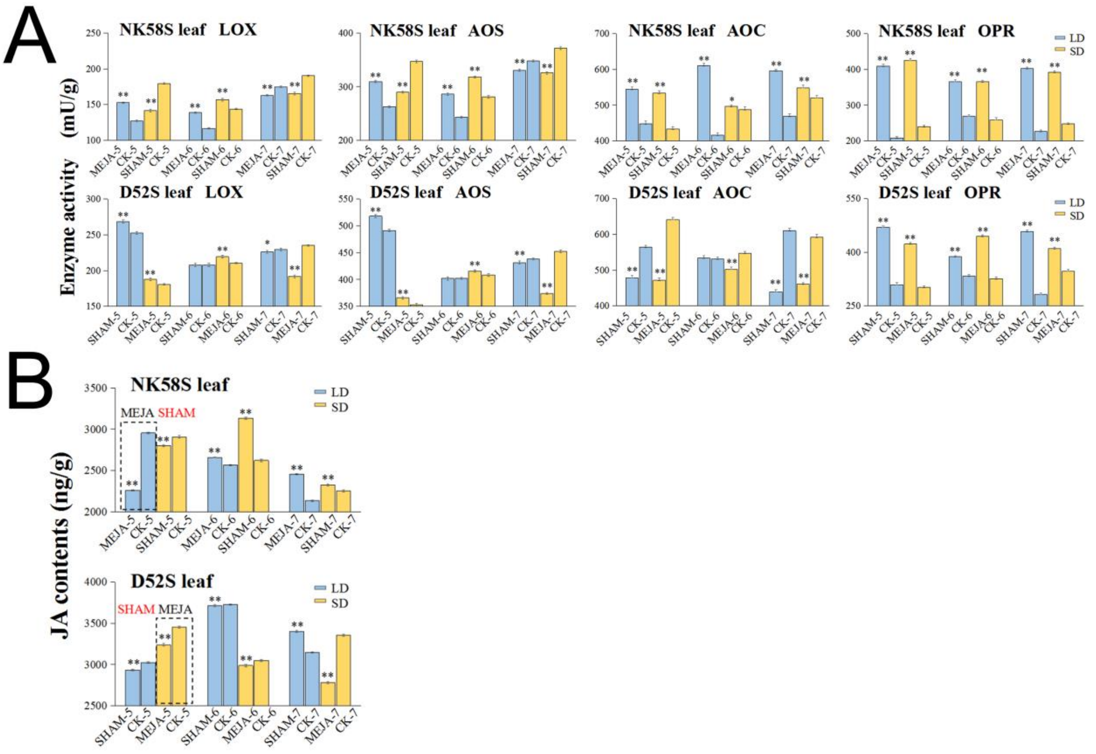

Figure S9. Analysis of JA synthesis enzyme activities and contents in D52S and NK58S by MEJA/SHAM-spraying treatment. The enzyme activities of LOX, AOS, AOC and OPR in the leaves from NK58S and D52S (A) and the JA contents (B) are also listed. The LD/SD treated plants are columnar with blue/yellow colour, respectively, the spraying plants was named CK/MEJA/SHAM in X-axis. Data are shown as mean ± standard deviation (n = 3). Asterisks indicate significant differences from spraying-induced fertile/sterile plants, compared to CK group, revealed by Student's *t*-test (*, $P < 0.05$; **, $P < 0.01$). LD, long-day cycle; SD, short-day cycle; MEJA, methyl jasmonate; SHAM, salicylhydroxamic acid; CK, control; 5/6/7, 5th/6th/7th-stage.

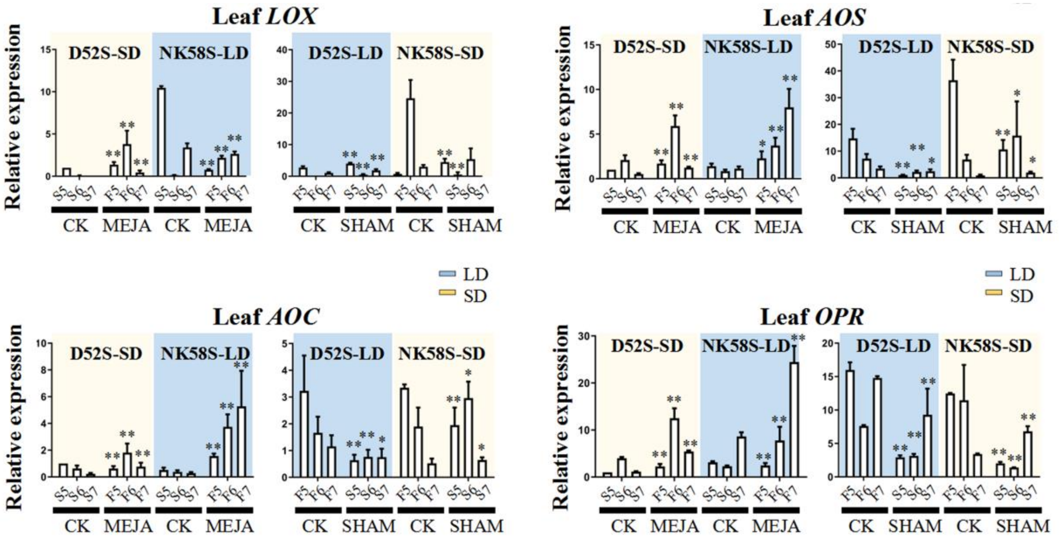

Figure S10. Expressions analysis of JA synthesis enzyme genes in the leaves of fertility-reverse plants treated by MEJA/SHAM-spraying treatment. The qPCR analysis of *LOX*, *AOS*, *AOC* and *OPR* in the leaves from NK58S and D52S sterile/fertile/fertility-reverse lines during 5th-7th-stage. The internal reference gene is *OsActin*. The LD/SD treated plants are columnar with blue/yellow colour, respectively, the spraying plants was named CK/MEJA/SHAM in X-axis. Data are shown as mean ± standard deviation (n = 3). Asterisks indicate significant differences from spraying-induced fertile/sterile plants, compared to CK group, revealed by Student's *t*-test (*, $P < 0.05$; **, $P < 0.01$). LD, long-day cycle; SD, short-day cycle; MEJA, methyl jasmonate; SHAM, salicylhydroxamic acid; CK, control; S/F/R, sterile/fertile/fertility-reverse plants; 5/6/7, $5^{th}/6^{th}/7^{th}$-stage.

Table S1. Sequencing data, statistics and genome comparison analysis from the 4th-stage spikelets of PA64S, NK58S and D52S.

Sample	Raw reads	Clean reads	Clean ratio [a]	All reads	Mapped reads	Mapped unique reads [b]	Mapping ratio [c]
PA64S-S	53790413	51210131	95.21	51024596	45697567	43481779	89.57
PA64S-F	46280604	43823754	94.65	43648077	38702350	36875397	88.59
D52S-S	42255637	40205882	95.16	39976556	35490375	33838260	88.78
D52S-F	43530215	41436642	95.19	41254937	36777892	34980002	89.15
NK58S-S	44577617	42372393	95.06	42187943	39741926	37882930	94.21
NK58S-F	47041822	44712212	95.06	44555801	41958612	39977882	94.19

For each sample, data are shown as mean by three biological replicates. [a] Clean ratio = (Clean reads/Raw reads) %; [b] Mapped unique reads, only one location matching reads in the genome. The reference genome information was obtained from the Ensemble database of *Oryza sativa* L. *japonica* (http://ensemblgenomes.org/, accessed in 2020); [c] Mapping ratio = Mapped reads/All reads.

Table S2. GO enrichment analysis of DEGs in the 4th-stage spikelets between fertile and sterile lines from PA64S, NK58S and D52S.

GO ID	GO Term	DEG	UP [a]	DOWN [b]	*P*-value	FDR [c]
			**PA64S**			
GO:0003964	RNA-directed DNA polymerase activity	1	1	0	1.26×10^{-17}	8.17×10^{-15}
GO:0006278	RNA-dependent DNA replication	1	1	0	1.45×10^{-16}	4.70×10^{-14}
GO:0003723	RNA binding	4	3	1	7.33×10^{-16}	1.59×10^{-13}
GO:0010200	response to chitin	32	30	2	2.12×10^{-13}	3.44×10^{-11}
GO:0003700	sequence-specific DNA binding transcription factor activity	49	44	5	1.86×10^{-12}	2.41×10^{-10}
GO:0009409	response to cold	28	25	3	2.12×10^{-12}	2.30×10^{-10}
GO:0009408	response to heat	20	20	0	5.95×10^{-12}	5.52×10^{-10}
GO:0009644	response to high light intensity	17	16	1	4.10×10^{-10}	3.33×10^{-8}
GO:0009611	response to wounding	24	23	1	6.28×10^{-10}	4.53×10^{-8}
GO:0042542	response to hydrogen peroxide	15	15	0	5.09×10^{-9}	3.30×10^{-7}
GO:0009753	**response to jasmonic acid**	17	16	1	5.80×10^{-9}	3.42×10^{-7}
GO:0009414	response to water deprivation	21	19	2	7.99×10^{-9}	4.32×10^{-7}
GO:0009738	**abscisic acid-activated signaling pathway**	17	17	0	1.10×10^{-8}	5.48×10^{-7}

GO:0009723	**response to ethylene**	19	19	0	1.16×10^{-8}	5.37×10^{-7}
GO:0035556	intracellular signal transduction	14	14	0	2.74×10^{-8}	1.18×10^{-6}
GO:0006508	proteolysis	2	2	0	3.01×10^{-8}	1.22×10^{-6}
GO:0042538	hyperosmotic salinity response	14	13	1	5.22×10^{-8}	1.99×10^{-6}
GO:0009620	response to fungus	11	11	0	6.09×10^{-8}	2.20×10^{-6}
GO:0009733	**response to auxin**	18	15	3	1.20×10^{-7}	4.10×10^{-6}
GO:0006355	regulation of transcription, DNA-templated	35	34	1	2.26×10^{-7}	7.32×10^{-6}
GO:0034976	response to endoplasmic reticulum stress	13	12	1	3.30×10^{-7}	1.02×10^{-5}
GO:0009695	**jasmonic acid biosynthetic process**	12	12	0	4.28×10^{-7}	1.26×10^{-5}
GO:0030968	endoplasmic reticulum unfolded protein response	13	13	0	4.39×10^{-7}	1.24×10^{-5}
GO:0032880	regulation of protein localization	6	6	0	1.52×10^{-6}	4.12×10^{-5}
GO:0016798	hydrolase activity, acting on glycosyl bonds	6	6	0	4.43×10^{-6}	1.15×10^{-4}
GO:0006457	protein folding	13	13	0	6.50×10^{-6}	1.62×10^{-4}
GO:0009266	response to temperature stimulus	9	8	1	7.74×10^{-6}	1.86×10^{-4}
GO:0016762	xyloglucan:xyloglucosyl transferase activity	4	4	0	8.00×10^{-6}	1.85×10^{-4}
GO:0006972	hyperosmotic response	8	7	1	1.28×10^{-5}	2.87×10^{-4}

GO:0009873	**ethylene-activated signaling pathway**	9	9	0	1.35×10^{-5}	2.91×10^{-4}
GO:0003677	DNA binding	37	31	6	1.45×10^{-5}	3.04×10^{-4}
GO:0046686	response to cadmium ion	17	15	2	1.72×10^{-5}	3.49×10^{-4}
GO:0005509	calcium ion binding	9	9	0	1.82×10^{-5}	3.57×10^{-4}
GO:0006833	water transport	9	8	1	1.82×10^{-5}	3.57×10^{-4}
GO:0010286	heat acclimation	6	6	0	1.87×10^{-5}	3.48×10^{-4}
GO:0048767	root hair elongation	11	10	1	2.08×10^{-5}	3.74×10^{-4}
GO:0006984	ER-nucleus signaling pathway	3	3	0	2.36×10^{-5}	4.15×10^{-4}
GO:0009651	response to salt stress	23	21	2	3.09×10^{-5}	5.28×10^{-4}
GO:0009646	response to absence of light	4	3	1	3.70×10^{-5}	6.16×10^{-4}
GO:0009867	**jasmonic acid mediated signaling pathway**	14	14	0	8.52×10^{-5}	1.23×10^{-3}
			**D52S**			
GO:0003723	RNA binding	4	2	2	1.18×10^{-28}	1.09×10^{-25}
GO:0009414	response to water deprivation	41	15	26	2.30×10^{-17}	1.06×10^{-14}
GO:0006508	proteolysis	2	0	2	1.37×10^{-14}	4.24×10^{-12}
GO:0009737	**response to abscisic acid**	40	13	27	2.19×10^{-13}	5.06×10^{-11}

GO:0042538	hyperosmotic salinity response	25	12	13	3.88×10^{-13}	7.18×10^{-11}
GO:0009409	response to cold	37	17	20	1.27×10^{-12}	1.96×10^{-10}
GO:0009753	**response to jasmonic acid**	24	16	8	2.96×10^{-10}	3.91×10^{-8}
GO:0009723	**response to ethylene**	27	13	14	7.08×10^{-10}	8.19×10^{-8}
GO:0003674	molecular function	150	43	107	1.09×10^{-9}	1.12×10^{-7}
GO:0009611	response to wounding	31	18	13	1.70×10^{-9}	1.57×10^{-7}
GO:0009733	**response to auxin**	27	18	9	1.91×10^{-9}	1.61×10^{-7}
GO:0009738	**abscisic acid-activated signaling pathway**	23	15	8	3.18×10^{-9}	2.45×10^{-7}
GO:0009535	chloroplast thylakoid membrane	23	2	21	4.86×10^{-9}	3.46×10^{-7}
GO:0015979	photosynthesis	14	1	13	5.16×10^{-9}	3.41×10^{-7}
GO:0009620	response to fungus	13	9	4	3.59×10^{-7}	2.22×10^{-5}
GO:0009579	thylakoid	16	1	15	5.05×10^{-7}	2.92×10^{-5}
GO:0006833	water transport	14	7	7	5.30×10^{-7}	2.89×10^{-5}
GO:0006260	DNA replication	10	9	1	7.88×10^{-7}	4.05×10^{-5}
GO:0009651	response to salt stress	36	19	17	1.33×10^{-6}	6.48×10^{-5}
GO:0009506	plasmodesma	41	26	15	1.51×10^{-6}	6.97×10^{-5}

GO:0008283	cell proliferation	13	13	0	3.04×10^{-6}	1.34×10^{-4}
GO:0009534	chloroplast thylakoid	14	1	13	4.58×10^{-6}	1.92×10^{-4}
GO:0009739	**response to gibberellin**	11	6	5	4.83×10^{-6}	1.94×10^{-4}
GO:0009631	cold acclimation	7	3	4	1.15×10^{-5}	4.44×10^{-4}
GO:0010200	response to chitin	27	19	8	1.27×10^{-5}	4.71×10^{-4}
GO:0009644	response to high light intensity	15	6	9	1.34×10^{-5}	4.78×10^{-4}
GO:0030076	light-harvesting complex	4	0	4	1.46×10^{-5}	5.00×10^{-4}
GO:0009695	**jasmonic acid biosynthetic process**	13	10	3	2.19×10^{-5}	7.22×10^{-4}
GO:0009750	response to fructose	11	5	6	3.18×10^{-5}	1.01×10^{-3}
GO:0006268	DNA unwinding involved in DNA replication	3	3	0	3.72×10^{-5}	1.15×10^{-3}
GO:0007165	signal transduction	18	14	4	5.44×10^{-5}	1.62×10^{-3}
GO:0035556	intracellular signal transduction	13	12	1	5.62×10^{-5}	1.62×10^{-3}
GO:0006612	protein targeting to membrane	24	18	6	5.68×10^{-5}	1.59×10^{-3}
GO:0070838	divalent metal ion transport	9	2	7	6.24×10^{-5}	1.70×10^{-3}
GO:0010363	regulation of plant-type hypersensitive response	24	18	6	6.37×10^{-5}	1.68×10^{-3}
GO:0009538	photosystem I reaction center	3	0	3	6.57×10^{-5}	1.69×10^{-3}

GO:0051726	regulation of cell cycle	9	9	0	8.42×10^{-5}	2.10×10^{-3}
GO:0001666	response to hypoxia	8	1	7	8.65×10^{-5}	2.11×10^{-3}
GO:0016161	beta-amylase activity	3	0	3	1.07×10^{-4}	2.55×10^{-3}
GO:0006275	regulation of DNA replication	8	8	0	1.20×10^{-4}	2.77×10^{-3}
GO:0010295	**(+)-abscisic acid 8'-hydroxylase activity**	2	0	2	5.41×10^{-5}	8.56×10^{-4}
GO:0009832	plant-type cell wall biogenesis	9	2	7	6.88×10^{-5}	1.06×10^{-3}
GO:0006612	protein targeting to membrane	17	2	15	7.44×10^{-5}	1.12×10^{-3}
GO:0010363	regulation of plant-type hypersensitive response	17	2	15	8.16×10^{-5}	1.20×10^{-3}
GO:0009867	**jasmonic acid mediated signaling pathway**	14	0	14	8.52×10^{-5}	1.23×10^{-3}
			**NK58S**			
GO:0009646	response to absence of light	3	3	0	1.00×10^{-6}	2.46×10^{-4}
GO:0009266	response to temperature stimulus	5	4	1	1.22×10^{-6}	1.50×10^{-4}
GO:0009644	response to high light intensity	6	6	0	1.43×10^{-6}	1.17×10^{-4}
GO:0006833	water transport	5	4	1	2.19×10^{-6}	1.34×10^{-4}
GO:0009408	response to heat	6	6	0	2.85×10^{-6}	1.40×10^{-4}
GO:0032880	regulation of protein localization	3	3	0	5.13×10^{-6}	2.10×10^{-4}

GO:0006457	protein folding	6	6	0	5.14×10^{-6}	1.80×10^{-4}
GO:0048574	long-day photoperiodism, flowering	2	1	1	9.21×10^{-6}	2.82×10^{-4}
GO:0006972	hyperosmotic response	4	3	1	$1.06 \times 10\text{-}^{5}$	2.89×10^{-4}
GO:0009739	**response to gibberellin**	4	3	1	1.12×10^{-5}	2.75×10^{-4}
GO:0042542	response to hydrogen peroxide	5	5	0	1.20×10^{-5}	2.67×10^{-4}
GO:0031225	anchored component of membrane	5	3	2	1.49×10^{-5}	3.04×10^{-4}
GO:0046686	response to cadmium ion	7	5	2	2.11×10^{-5}	3.97×10^{-4}
GO:0009733	**response to auxin**	6	5	1	3.46×10^{-5}	6.06×10^{-4}
GO:0050113	inositol oxygenase activity	1	0	1	4.60×10^{-5}	7.52×10^{-4}
GO:0019370	leukotriene biosynthetic process	1	1	0	4.60×10^{-5}	7.52×10^{-4}
GO:0018131	oxazole or thiazole biosynthetic process	1	1	0	4.60×10^{-5}	7.52×10^{-4}
GO:0080164	regulation of nitric oxide metabolic process	1	1	0	4.60×10^{-5}	7.52×10^{-4}
GO:0002540	leukotriene production involved in inflammatory response	1	1	0	4.60×10^{-5}	7.52×10^{-4}
GO:0009612	response to mechanical stimulus	3	3	0	4.76×10^{-5}	5.84×10^{-4}
GO:0045176	apical protein localization	1	0	1	9.19×10^{-5}	1.07×10^{-3}
GO:0042754	negative regulation of circadian rhythm	1	0	1	9.19×10^{-5}	1.32×10^{-3}

GO:0010038	response to metal ion	1	1	0	9.19×10^{-5}	1.32×10^{-3}
GO:0051592	response to calcium ion	1	1	0	9.19×10^{-5}	1.32×10^{-3}
GO:0005528	FK506 binding	2	2	0	1.06×10^{-4}	1.04×10^{-3}
GO:0000413	protein peptidyl-prolyl isomerization	2	2	0	1.06×10^{-4}	1.44×10^{-3}
GO:0018208	peptidyl-proline modification	2	2	0	1.06×10^{-4}	1.44×10^{-3}
GO:0009687	**abscisic acid metabolic process**	1	1	0	1.53×10^{-4}	1.34×10^{-3}
GO:0009228	thiamine biosynthetic process	1	1	0	1.53×10^{-4}	1.97×10^{-3}
GO:0009611	response to wounding	6	6	0	1.58×10^{-4}	1.29×10^{-3}
GO:0009753	**response to jasmonic acid**	3	2	1	2.29×10^{-4}	1.81×10^{-3}
GO:0009930	longitudinal side of cell surface	1	0	1	2.29×10^{-4}	2.67×10^{-3}
GO:0048767	root hair elongation	4	3	1	2.64×10^{-4}	1.96×10^{-3}
GO:0007030	Golgi organization	4	3	1	3.18×10^{-4}	2.29×10^{-3}
GO:0010215	cellulose microfibril organization	1	0	1	3.20×10^{-4}	2.24×10^{-3}
GO:0009695	**jasmonic acid biosynthetic process**	1	1	0	3.20×10^{-4}	3.27×10^{-3}
GO:0048827	phyllome development	1	0	1	3.20×10^{-4}	3.27×10^{-3}
GO:0006096	glycolytic process	4	3	1	3.44×10^{-4}	2.22×10^{-3}

GO:0030243	cellulose metabolic process	2	1	1	4.20×10^{-4}	2.64×10^{-3}
GO:0045300	acyl-[acyl-carrier-protein] desaturase activity	1	0	1	4.26×10^{-4}	2.61×10^{-3}

It is showing the top forty GO terms from PA64S, NK58S or D52S, which related to hormone are marked with red colour. [a] Number of significantly up-regulated genes in the 4th-stage spikelets compared with fertile and sterile plants; [b] Number of significantly down-regulated genes in the 4th-stage spikelets compared with fertile and sterile plants; [c] FDR is less than or equal to 0.05 as the threshold to define as GO terms significantly enriched in DEGs. GO, Gene Ontology; DEGs, differentially expressed genes; FDR, modified *p*-value.

Table S3. A Primer information of candidate genes related to JA biosynthesis.

Gene name	Raplocus	5'-3'
OsLOX	Os03g0700700	CAGCAAGATCACCGAAGCTC
		AGAATGGTCCTGCTCGCATA
OsAOS1	Os03g0767000	GCTCTTCCTCCGATACGACTC
		GCCTTCTTGAGCGAGGTGA
OsAOC3	Os03g0438100	TGTTTCCTCCCTGCGATGAG
		GTAGTGGCGGTCGTTGTAGT
OsOPR7	Os08g0459600	CATTGAGATCCATGGTGCTC
		CTGAATCATAGGCGTCAAGG
